# Development and Validation of a Machine Learning Model for Early Prediction of Delirium in Intensive Care Units Using Continuous Physiological Data: Retrospective Study

**DOI:** 10.2196/59520

**Published:** 2025-04-02

**Authors:** Chanmin Park, Changho Han, Su Kyeong Jang, Hyungjun Kim, Sora Kim, Byung Hee Kang, Kyoungwon Jung, Dukyong Yoon

**Affiliations:** 1 Department of Biomedical Systems Informatics Yonsei University College of Medicine Seoul Republic of Korea; 2 BUD.on Inc. Seoul Republic of Korea; 3 Ajou University Hospital Gyeonggi South Regional Trauma Center Suwon Republic of Korea; 4 Department of Surgery Division of Trauma Surgery Ajou University School of Medicine Suwon Republic of Korea

**Keywords:** delirium, intensive care unit, machine learning, prediction model, early prediction

## Abstract

**Background:**

Delirium in intensive care unit (ICU) patients poses a significant challenge, affecting patient outcomes and health care efficiency. Developing an accurate, real-time prediction model for delirium represents an advancement in critical care, addressing needs for timely intervention and resource optimization in ICUs.

**Objective:**

We aimed to create a novel machine learning model for delirium prediction in ICU patients using only continuous physiological data.

**Methods:**

We developed models integrating routinely available clinical data, such as age, sex, and patient monitoring device outputs, to ensure practicality and adaptability in diverse clinical settings. To confirm the reliability of delirium determination records, we prospectively collected results of Confusion Assessment Method for the ICU (CAM-ICU) evaluations performed by qualified investigators from May 17, 2021, to December 23, 2022, determining Cohen κ coefficients. Participants were included in the study if they were aged ≥18 years at ICU admission, had delirium evaluations using the CAM-ICU, and had data collected for at least 4 hours before delirium diagnosis or nondiagnosis. The development cohort from Yongin Severance Hospital (March 1, 2020, to January 12, 2022) comprised 5478 records: 5129 (93.62%) records from 651 patients for training and 349 (6.37%) records from 163 patients for internal validation. For temporal validation, we used 4438 records from the same hospital (January 28, 2022, to December 31, 2022) to reflect potential seasonal variations. External validation was performed using data from 670 patients at Ajou University Hospital (March 2022 to September 2022). We evaluated machine learning algorithms (random forest [RF], extra-trees classifier, and light gradient boosting machine) and selected the RF model as the final model based on its performance. To confirm clinical utility, a decision curve analysis and temporal pattern for model prediction during the ICU stay were performed.

**Results:**

The κ coefficient between labels generated by ICU nurses and prospectively verified by qualified researchers was 0.81, indicating reliable CAM-ICU results. Our final model showed robust performance in internal validation (area under the receiver operating characteristic curve [AUROC]: 0.82; area under the precision-recall curve [AUPRC]: 0.62) and maintained its accuracy in temporal validation (AUROC: 0.73; AUPRC: 0.85). External validation supported its effectiveness (AUROC: 0.84; AUPRC: 0.77). Decision curve analysis showed a positive net benefit at all thresholds, and the temporal pattern analysis showed a gradual increase in the model scores as the actual delirium diagnosis time approached.

**Conclusions:**

We developed a machine learning model for delirium prediction in ICU patients using routinely measured variables, including physiological waveforms. Our study demonstrates the potential of the RF model in predicting delirium, with consistent performance across various validation scenarios. The model uses noninvasive variables, making it applicable to a wide range of ICU patients, with minimal additional risk.

## Introduction

### Background

Delirium is a complex neuropsychiatric syndrome primarily characterized by fluctuations in consciousness and orientation, including alterations in the perception of dates, places, and persons [1,2], due to an imbalance in neurotransmitter levels or brain inflammation. Delirium is often triggered by medical illnesses, medications, or environmental factors such as sleep deprivation, sensory deprivation, and exposure to unfamiliar surroundings. Patients admitted to the intensive care unit (ICU) may develop overall cognitive function disorders (eg, impairment in attention or language skills) and psychotic disorders.

Delirium is associated with various adverse outcomes, significantly impacting patient health and health care systems. A meta-analysis by Witlox et al [3] found that delirium in older patients was associated with an increased risk of death (odds ratio 1.95), institutionalization (odds ratio 2.41, 95% CI, 1.77-3.29), and dementia (odds ratio 12.52, 95% CI, 1.86-84.21). Moreover, a study by Ely et al [4] reported that ICU patients with delirium had a 3.2 times higher 6-month mortality rate compared with those without delirium. Financially, Vasilevskis et al [5] estimated that delirium increases ICU costs by US $17,838 to US $24,584 per patient. Patients with delirium are at a higher risk for complications, such as falls, infections, and pressure ulcers [6]. In addition, given the complexity of the conditions of patients with delirium, health care providers caring for them may experience burnout and job dissatisfaction [7].

Given these significant impacts, early identification and prevention of delirium are crucial for reducing the burden on patients and health care systems [8]. Several prediction tools have been developed for this purpose, including PRE-DELIRIC (prediction model for delirium) and E-PRE-DELIRIC (early prediction model for delirium). PRE-DELIRIC, using data from the first 24 hours of ICU admission, has shown good discriminative ability (area under the receiver operating characteristic curve [AUROC]: 0.87) [9]. The E-PRE-DELIRIC model, usable within 2 hours of ICU admission, demonstrated similar performance (AUROC: 0.76) [10].

Machine learning approaches offer several potential advantages in delirium prediction. They have the ability to handle complex, nonlinear relationships between variables [11]; the capacity to process high-dimensional data, potentially uncovering subtle patterns not apparent in traditional statistical analyses [12]; and the flexibility to update predictions in real time as new data becomes available [13]. Although the superiority of machine learning models over traditional scoring systems in some critical care settings is not universally established [14,15], their potential in delirium prediction remains promising. Studies have demonstrated machine learning-based models’ efficacy in predicting delirium across various patient populations, including postoperative, older hip-arthroplasty, and patients with extensive burn [16-18]. These models, using diverse clinical parameters, have achieved high accuracy (AUROC: 0.84-0.94) [19,20].

Despite numerous attempts to predict delirium onset and prognosis, significant limitations persist. Missing data and inconsistent availability of dynamic measures across patients and settings further hinder model applicability [21,22]. In addition, real-time inference in clinical settings remains problematic. The difficulty in ensuring that variables reflect the current patient state, coupled with infrequent and inconsistent timing of data collection, impedes real-time monitoring and decision-making in fast-paced clinical environments [23]. Moreover, most existing models rely on static data points, failing to capture the dynamic nature of a patient’s condition.

Recent studies have highlighted the potential of machine learning models using routinely collected ICU data to enhance patient outcome predictions and clinical decision-making [24,25]. Existing delirium prediction models have primarily relied on static variables, whereas the incorporation of continuous data streams has shown promise in improving prediction accuracy in other ICU applications. For instance, Castiñeira et al [26] demonstrated that including continuous vital sign data significantly enhanced prediction accuracy for prolonged intubation stays. Similarly, Shickel et al [27] reported improved patient health predictions when combining routinely collected variables with novel data sources. These findings suggest that a delirium prediction model using continuous variables routinely collected in the ICU could offer substantial improvements in accuracy, timeliness, and clinical relevance compared with existing static variable-based models.

A model that uses only variables routinely monitored in ICUs should be developed to obtain real-time inferences. Electrocardiogram (ECG), photoplethysmogram (PPG), and respiratory waveforms are particularly suitable for this purpose. These noninvasive continuously monitored data streams are not only rich in physiological information but also readily available for most patients in ICUs.

Delirium is related to autonomic nervous system instability [28], which can cause changes in physiological signals. In particular, it is associated with changes in heart rate and blood pressure [29]. Moreover, autonomic nervous system instability affects heart rate variability (HRV); thus, HRV serves as an effective predictor of delirium [30]. A prospective cohort study reported the association between HRV and delirium [31], and a recent study showed that delirium could be predicted using HRV estimated from an ECG [32]. Both the PPG and ECG are typically used to estimate the HRV [33,34], and respiratory waveforms and rates can be incorporated as model inputs, considering their clinical relevance [35].

Unlike previous studies that relied on static clinical variables, our approach focuses on continuous, high-frequency data from ECG, PPG, and respiratory waveforms. This dynamic data capture may allow our model to detect subtle physiological changes preceding delirium onset, which static models might miss. Moreover, the ability of machine learning models to continuously update predictions based on incoming data aligns well with the fluctuating nature of delirium symptoms [30].

### Objectives

This study aimed to create a novel machine learning model for real-time delirium prediction in ICU patients using only routinely monitored variables (ECG, PPG, and respiratory waveforms).

On the basis of these insights and our research objectives, we hypothesized that (1) a machine learning model using readily available, continuously monitored signals (ECG, PPG, and respiratory waveforms) can effectively predict the onset of delirium in ICU patients; (2) this model will demonstrate comparable performance with existing prediction methods, despite relying on a more focused set of routinely collected data rather than complex or less accessible variables; (3) the model’s performance will remain consistent across different patient populations and time frames, as demonstrated through temporal and external validation; and (4) by leveraging continuous data streams, the model will capture subtle physiological changes preceding delirium onset, potentially enabling earlier detection and intervention, compared with models using static variables.

By testing these hypotheses, we aimed to develop a practical, real-time tool for delirium prediction that can be easily integrated into various ICU settings, potentially improving early detection and management of this critical condition. This approach aligns with recent advancements in ICU-based machine learning models and addresses the need for more dynamic, widely applicable prediction tools in critical care.

## Methods

### Ethical Considerations

This study was conducted in accordance with ethical research principles and was approved by the institutional review board (IRB) of Yongin Severance Hospital. The need for informed consent for the use of retrospective data was waived (9-2021-0032); however, prospective data collection was performed after obtaining informed consent from the patients (9-2021-0186). In addition, the need for informed consent for the use of temporal validation data was waived (9-2024-0023). For the external validation cohort, ethics approval was obtained from the IRB of Ajou University Hospital (AJOUIRB-OBS-2021-084).

We followed the guidelines for developing and reporting machine learning predictive models in biomedical research and the Transparent Reporting of a multivariable prediction model for Individual Prognosis or Diagnosis+Artificial Intelligence (TRIPOD+AI) guidelines for transparent reporting [36,37]. The reporting checklists are available in Multimedia Appendix 1 [38-41].

### Data Collection and Study Population

Data were collected from 2 health care institutions, namely, Yongin Severance Hospital and Ajou University Hospital. The collected data included electronic medical records from both hospitals to construct a comprehensive dataset.

Patient inclusion criteria are shown in Textbox 1.

**Patient inclusion criteria.**

- Aged ≥18 years on the day of intensive care unit admission
- Evaluated for delirium using the Confusion Assessment Method for the ICU
- Having data collected for at least 4 hours before the time of delirium diagnosis or nondiagnosis

Data from Yongin Severance Hospital were obtained both retrospectively and prospectively. Retrospective data for model training and internal validation were collected from March 1, 2020, to January 12, 2022, whereas data for temporal validation were obtained from January 28, 2022, to December 31, 2022, which covers nearly a year. To account for potential seasonal variations in delirium, the temporal validation set was divided into fixed quarterly intervals: Q1 (January-March), Q2 (April-June), Q3 (July-September), and Q4 (October-December). This approach enhances the model’s robustness over time by performance across different periods and ensures adaptability to evolving patient demographics ant treatment practices.

Results of the Confusion Assessment Method for the ICU (CAM-ICU) were collected and analyzed independently and prospectively from May 17, 2021, to December 23, 2022 for verification by ICU nurses. For external validation, data from Ajou University Hospital, which is geographically distinct from the development institution, were retrospectively obtained from March to September 2022. Patients aged <18 years and those without CAM-ICU records were excluded from data analysis.

### Prediction Variables and Outcome

Our model used features derived from physiological signals and basic patient demographics. The variables were selected based on their potential relevance to delirium prediction, their continuous availability in ICU settings, and their noninvasive nature, ensuring broad applicability across ICU patients.

The features included demographic variables (age and sex) and parameters derived from ECG lead II, PPG, and respiratory waveforms. Specifically, we extracted Hjorth parameters (activity, complexity, and mobility), which provide information about the signal’s time domain properties [42], as well as kurtosis and skewness, which can capture abnormalities in waveform morphology [43]. In addition, we incorporated vital signs, including heart rate, respiratory rate, and oxygen saturation (SpO_2_), represented by their median and SD.

The outcome variable was the occurrence of delirium, as determined by the CAM-ICU assessment.

### CAM-ICU Evaluation and Reliability Verification

**Figure 1:**
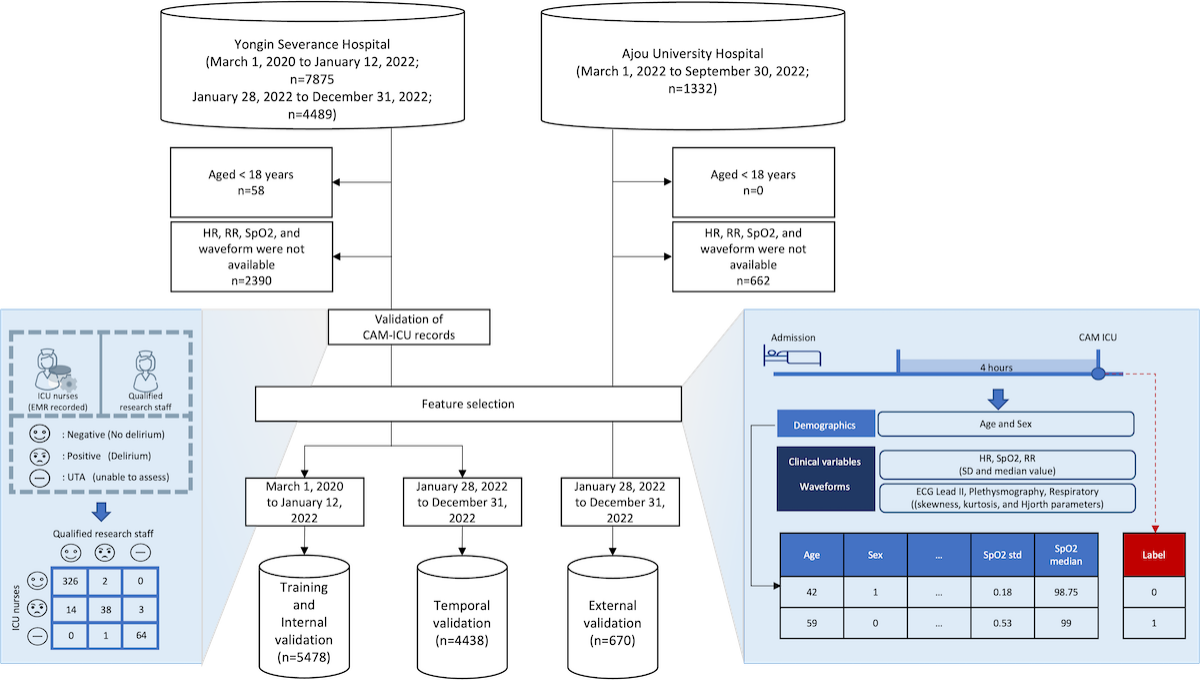
Development and validation process of the delirium prediction model in 2 hospitals. This flowchart illustrates the process of training the machine learning model with patients’ data (including age, sex, vital signs, and waveforms) from Yongin Severance Hospital, temporal validation of the model, and external validation using data from Ajou University Hospital. The results of 2 independent raters are compared with confirm the reliability of Confusion Assessment Method for the ICU (CAM-ICU) scores. EMR: electronic medical record; HR: heart rate; RR: respiratory rate; SpO2: oxygen saturation.

The reliability of CAM-ICU results documented in electronic medical records was examined. CAM-ICU evaluations were conducted and verified independently by qualified registered nurses, adhering to the Vanderbilt CAM-ICU training manual guidelines [44]. The agreement between the results obtained by research staff and those generated by ICU nurses was evaluated using Cohen κ statistic (Figure 1) [45].

### Feature Extraction and Data Preprocessing

We extracted features from physiological waveform data (ECG lead II, PPG, and respiratory waveforms). Before feature extraction, we implemented a comprehensive noise removal process, eliminating characteristic patterns indicative of sensor failure or device error (Multimedia Appendix 1). Following noise removal, waveforms were preprocessed by normalization (Multimedia Appendix 2).

To mitigate the impact of extreme values and account for the inherently noisy nature of physiological signals, we calculated representative values (medians and SDs) over a 4-hour predictive window before delirium assessment for each feature (Multimedia Appendix 3). This approach ensures robust model operation by minimizing the influence of outliers.

To maximize data integrity, we excluded any data with missing values. This rigorous preprocessing and standardization pipeline was applied consistently to data from both health care institutions, ensuring a harmonized dataset for model development.

### Algorithm Selection

We focused on tree-based models for our delirium prediction task due to their ability to handle complex, nonlinear relationships and their built-in methods for assessing feature importance. We evaluated the performances of 3 specific tree-based models: the random forest (RF) classifier [46], extra-trees classifier [47], and light gradient boosting model (LightGBM) [48].

In selecting our final model, we prioritized the AUROC. The AUROC offers several advantages: it is threshold-independent, providing a comprehensive evaluation across all possible classification thresholds [49], and demonstrates robustness to class imbalance [50]. These characteristics are particularly valuable in clinical settings where optimal intervention thresholds may vary, and class distributions can be uneven. The AUROC also allows for direct comparison between different models across varying datasets [51], which is crucial in clinical research involving diverse patient populations.

Our emphasis on the AUROC is supported by research demonstrating its robustness in imbalanced datasets, common in clinical scenarios such as delirium prediction. Bekkar et al [52] highlighted AUROC’s stability across different class distributions, whereas Jeni et al [53] emphasized its advantage in providing an aggregate measure of performance across all classification thresholds. These attributes align with our goal of developing a flexible model adaptable to various clinical settings. The AUROC guided our model selection, and we also report additional metrics for a comprehensive evaluation of our model’s capabilities.

### Model Development

We used an automated machine learning workflow to streamline our model development process. This approach included several key steps:

1. Data preparation—we preprocessed the dataset by handling missing values, encoding categorical variables, and normalizing features. The data were split into training and validation sets to ensure that our model would be evaluated on unseen data.
2. Initial model training—we trained an initial RF model using default hyperparameters to establish baseline performance.
3. Addressing class imbalance—given the nature of delirium occurrence in ICU settings, our dataset exhibited an imbalance between delirium and nondelirium cases. To address this imbalance and ensure our model’s performance was not biased toward the majority class, we implemented several strategies in our RF (Multimedia Appendix 1).

After optimizing the hyperparameter, the model was trained using the entire training cohort. This phase also included internal validation to refine and adjust the prediction model. Data for training and internal validation were carefully divided at the patient level at an 8:2 ratio, while ensuring that records from the same patients were not repeated between the datasets. The development cohort comprised 5478 records, with 5129 (93.62%) records from 651 patients allocated for training and 349 (6.37%) records from 163 patients allocated for internal validation.

### Model Validation

In addition to the aforementioned internal validation, further validation efforts were expended, including both temporal and external validations. The temporal validation approach involved an analysis of data from a distinct period not used during the model’s initial development phase. This strategy was deliberately selected to ensure that the dataset for validation did not overlap with the dataset for development, thereby enhancing the generalizability and relevance of the model in real-world settings. For this purpose, 4438 records from ICUs were identified using a time frame different from that of the initial model training. Such temporal validation presented substantial benefits. First, it enabled the evaluation of the model’s ability to adapt to changes in clinical patterns, patient demographics, or treatment protocols over time, which is essential in the ever-evolving health care sector. Second, testing the model with data from various periods aids in diminishing the risk of overfitting to the specific characteristics of the initial training set, thereby ensuring a more dependable and robust model. Third, this method is particularly aligned with ongoing developments in medical practices and patient care, providing insights into the model’s long-term performance.

Data from Ajou University Hospital were used for external validation. Initially, a cohort of 1332 patients was screened for delirium; of these patients, 670 (50.3%) were selected for inclusion after a detailed review process. The meticulous selection bolstered the robustness and reliability of the model by omitting cases that lacked complete clinical data or had missing waveform data. The implementation of such rigorous exclusion criteria was imperative to ensure that our analysis was conducted on the most accurate and comprehensive dataset possible and to confirm the validity of the model in a practical clinical environment.

### Decision Curve Analysis

A decision curve analysis [54] was performed to assess the clinical utility of our prediction model. The primary concept in decision curve analysis is “net benefit,” which compares the advantage of correct positive predictions to the disadvantage of false positive predictions, weighted by the threshold probability.

The threshold probability is representing the point at which the potential benefits or treatment equal the potential risks. For example, a threshold probability of 0.2 suggests that treatment would be recommended if a patient has a 20% or greater probability of developing the condition (in this case, delirium).

We plotted the net benefit of our prediction model across a range of threshold probabilities and compared it with 2 baseline strategies: “treat all” (assume all patients will develop delirium) and “treat none” (assume no patients will develop delirium). These strategies represent the extremes of clinical decision-making and provide a context for evaluating our model’s performance. The “treat all” strategy corresponds to a threshold probability of 0, whereas “treat none” corresponds to a threshold probability of 1.

Our model is considered clinically useful if it demonstrates a higher net benefit than both baseline strategies across a range of clinically relevant threshold probabilities. This approach helps to determine the range of threshold probabilities where using our model’s predictions would lead to better clinical decisions than uniformly treating all patients or no patients.

The primary parameter we aimed to optimize in this analysis was the net benefit of our model across different threshold probabilities. By comparing our model’s net benefit to the baseline strategies, we can identify the range of threshold probabilities where our model provides the most clinical value in guiding delirium prevention or early intervention strategies (Multimedia Appendix 1).

### Statistical Analysis

We used statistical methods to analyze our data and evaluate model performance. To compare categorical variables between groups, we used chi-square tests. Continuous variables were compared using 2-tailed independent *t* tests for normally distributed data or Mann-Whitney *U* tests for nonnormally distributed data. Normality was assessed using the Shapiro-Wilk test.

In addition, we analyzed correlations so that we could intuitively examine the relationship between each variable we considered and the outcome.

The AUROC, sensitivity, positive predictive value, and accuracy at a threshold were measured to compare the performance of different models. Data processing was performed using Python version 3.6.13. The machine learning model was developed and validated using the PyCaret library version 2.3.10.

## Results

### Population Characteristics

The training and internal validation cohorts comprised 5478 CAM-ICU records. The temporal validation cohort consisted of 4438 CAM-ICU records, whereas the external validation cohort consisted of 670 CAM-ICU records. Each cohort comprised distinct patient populations.

Table 1 summarizes the baseline characteristics of the training and validation cohorts. The mean age was 65.9 (SD 15.6) years in the training and internal validation sets, 70.2 (SD 15.7) years in the temporal validation set, and 58.6 (SD 21.3) years in the external validation set, with a significant difference in age distribution among the cohorts (*P*<.001). In addition to age, the cohorts exhibited significant differences in sex and other variables used as model inputs. The internal validation set and temporal validation set included both medical and surgical patients from general hospital ICUs, whereas the external validation set included patients from only a trauma ICU.

**Table 1:** Characteristics of datasets from the 2 hospitals.

		Training and internal validation sets	Temporal validation set	External validation set
Patients, n		5478	4438	670
**Patients per room, n (%)**				
	Medical ICU^a^	3160 (57.7)	2734 (62.1)	—^b^
	Surgical ICU	2318 (42.3)	1667 (37.9)	—
	Trauma ICU	—	—	670 (100.0)
	Missing	—	37 (0.8)	—
Primary outcome ratio, n		0.6	0.7	0.4
**Sex, n (%)**				
	Male	3203 (58.5)	2619 (59.0)	582 (86.9)
	Female	2275 (41.5)	1819 (41.0)	88 (13.1)
Age (y), mean (SD)		66.0 (15.5)	70.2 (15.7)	58.6 (21.3)
ECG^c^ lead II (Hjorth activity), mean (SD)		4.2 (10.0)	3.0 (4.3)	4.0 (3.8)
PPG^d^ (Hjorth activity), mean (SD)		0.5 (1.2)	0.5 (0.9)	0.5 (0.2)
Respiratory waveform (Hjorth activity), mean (SD)		0.4 (0.2)	0.5 (0.3)	0.6 (0.4)
ECG lead II (Hjorth complexity), mean (SD)		2.3 (0.8)	4.4 (0.9)	1.7 (0.2)
ECG lead II (Hjorth mobility), mean (SD)		0.4 (0.1)	0.3 (0.1)	0.3 (0.0)
ECG lead II (kurtosis), mean (SD)		15.9 (14.8)	9.6 (6.1)	11.3 (6.2)
PPG (kurtosis), mean (SD)		−0.7 (0.5)	−0.3 (0.9)	−0.4 (1.0)
Respiratory waveform (kurtosis), mean (SD)		−1.0 (0.4)	−0.5 (1.5)	−0.1 (0.8)
ECG lead II (skewness), mean (SD)		2.8 (1.9)	2.1 (1.4)	2.8 (1.3)
PPG (skewness), mean (SD)		0.4 (0.3)	0.4 (0.3)	0.5 (0.3)
Respiratory waveform (skewness), mean (SD)		0.4 (0.3)	0.4 (0.3)	0.9 (0.3)
HR^e^ (median), mean (SD)		81.6 (17.6)	83.5 (17.1)	90.1 (18.1)
RR^f^ (median), mean (SD)		18.2 (4.6)	18.8 (4.4)	18.9 (5.3)
SpO_2_^g^ (median), mean (SD)		98.8 (1.6)	98.7 (2.1)	99.1 (1.4)
HR (SD), mean (SD)		5.1 (4.3)	4.8 (3.3)	4.6 (2.7)
RR (SD), mean (SD)		2.3 (1.1)	2.5 (1.1)	3.7 (2.1)
SpO_2_ (SD), mean (SD)		0.5 (0.6)	1.1 (1.7)	0.9 (0.9)

^a^ICU: intensive care unit.

^b^Not applicable.

^c^ECG: electrocardiogram.

^d^PPG: photoplethysmogram.

^e^HR: heart rate.

^f^RR: respiratory rate.

^g^SpO_2_: oxygen saturation

### Reliability of CAM-ICU Results

A comparative analysis between the outcomes recorded by ICU nurses and those independently evaluated by the research staff was performed to evaluate the reliability of predictive CAM-ICU results. Furthermore, the degree of agreement between these 2 types of observations was quantified using Cohen κ (Table 2). The κ coefficient was 0.81, indicating a high agreement between observers. This robust agreement implied the reliability of predicted CAM-ICU results and validated our findings.

**Table 2:** Agreement between intensive care unit nurses and qualified research staff.

	Negative	Positive	Unable to assess
Negative	326	2	0
Positive	14	38	3
Unable to assess	0	1	64

### Model Selection

Among the tree-based models evaluated (RF, extra-trees, and LightGBM), all 3 models showed competitive performance across various metrics (Table 3). However, the RF classifier demonstrated slightly superior performance in key areas.

The RF model achieved the highest AUROC of 0.757, indicating the best overall discriminative ability across all possible classification thresholds. This marginally outperformed the extra-trees (AUROC: 0.748) and LightGBM (AUROC: 0.745). In terms of precision, the RF model excelled, with a score of 0.725, which was slightly higher than those of the extra-trees (0.724) and LightGBM (0.720). This higher precision indicates a lower false positive rate, which is crucial in clinical settings to avoid unnecessary interventions.

**Table 3:** Performance of tree-based models before hyperparameter tuning.

Model	Accuracy	Area under the curve	Recall	Precision	*F*_1_- score	κ coefficient	Matthew correlation coefficient	Training time (seconds)
RF^a^	0.682	*0.757* ^b^	0.687	0.725	0.701	0.361	0.366	0.317
Extra-trees classifier	0.687	0.748	0.704	0.724	0.711	0.370	0.373	0.210
LightGBM^c^	0.672	0.745	0.670	0.720	0.689	0.342	0.347	0.053

^a^RF: random forest.

^b^Italics indicate the model with the best performance in the algorithm selection process.

^c^LightGBM: light gradient boosting model.

In addition, after applying our model’s development process to each benchmark model and comparing AUROC and area under the precision-recall curve (AUPRC), our model showed the best performance (Multimedia Appendix 4). On the basis of these results, particularly the superior area under the curve and precision scores, the RF classifier was selected as our final model for delirium prediction. This selection aligns with our methodology of prioritizing overall discriminative ability and minimizing false positives in clinical applications, while also considering the balance between precision and recall as reflected in the *F*_1_- score.

### Model Performance

**Figure 2:**
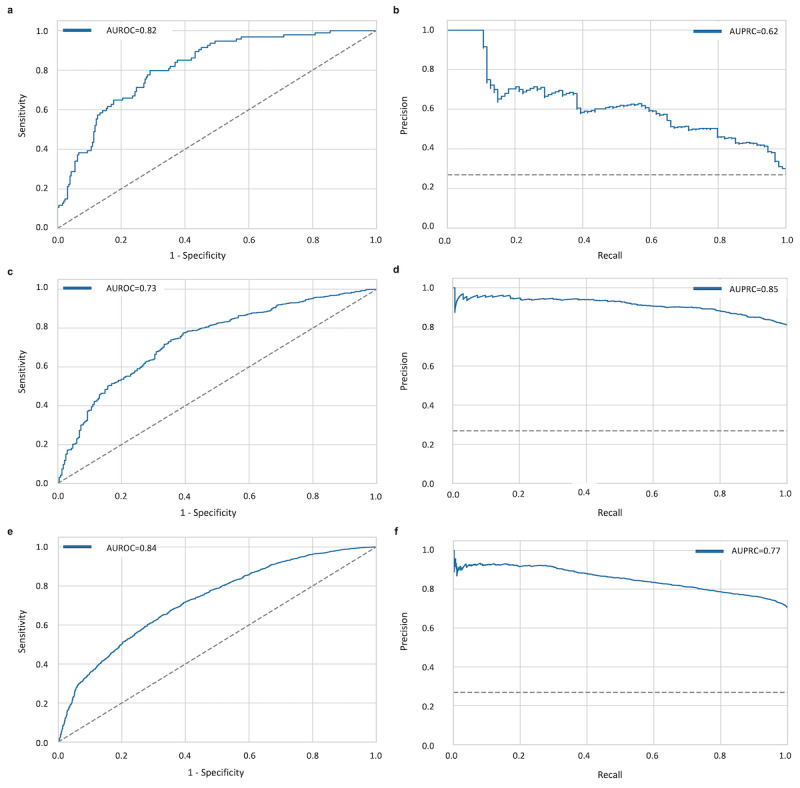
Model performance in the internal and external validation cohorts. The performance of the developed model in the internal and external validation cohorts is shown. (A) The area under the receiver operating characteristic curve (AUROC) and (B) area under the precision-recall curve (AUPRC) of the model in the internal validation cohort. (C) The AUROC and (D) AUPRC of the model in the temporal validation cohort. (E) The AUROC and (F) AUPRC of the model in the external validation cohort.

In the internal validation cohort, the RF showed strong performance, with an AUROC of 0.82 and an AUPRC of 0.62 for the overall cohort (Figure 2). The model achieved an AUROC of 0.73 and an AUPRC of 0.85 in the temporal validation cohort, where consistent performance metrics were confirmed across quarterly periods (Q1-Q4; Multimedia Appendix 5), and an AUROC of 0.82 and AUPRC of 0.77 in the external validation cohort, indicating its robustness and generalizability. These results suggested that the model effectively discriminated between delirium and nondelirium cases, highlighting its predictive capabilities.

In addition, the correlation coefficient between each variable and the outcome did not have a high correlation coefficient overall but had the highest values for age, PPG-derived variables, and SpO_2_-derived variables (Multimedia Appendix 6).

**Figure 3:**
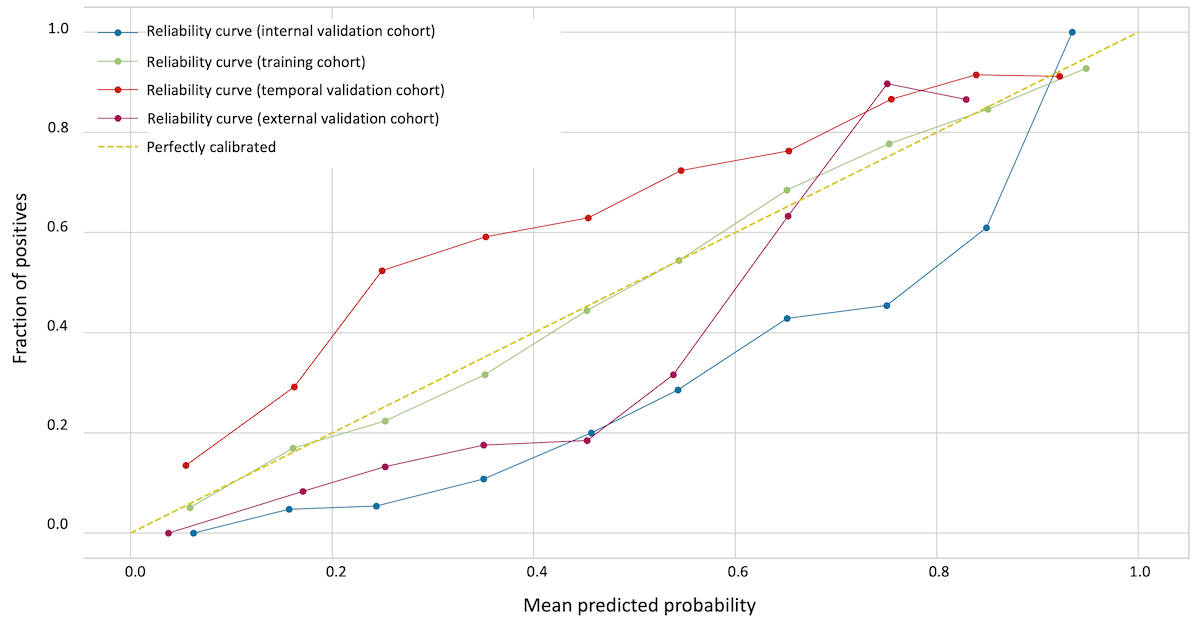
Calibration curve showing the reliability of our delirium prediction model. Calibration curve for the training (blue line), internal validation (green line), and external validation (red line) datasets. The x-axis represents the predicted probability of delirium as output by our model, whereas the y-axis represents the observed frequency of delirium in the validation cohorts. The black dashed line indicates perfect calibration, in which the predicted probabilities exactly match the observed outcomes.

A thorough analysis focusing on the calibration curves and alignment between predicted probabilities and observed outcomes was conducted to assess the reliability of the prediction model. Figure 3 presents the model’s unfitted calibration curves on 4 distinct datasets (namely, the training, internal validation, temporal validation, and external validation datasets), with the curves showing the concordance between the model’s estimated probability for the positive class and the actual occurrence of that class. In the training cohort, the calibration curve (green line) adhered closely to the ideal calibration line (dotted line), suggesting that the model’s predicted probabilities strongly agreed with the observed outcomes in the training dataset. In the internal validation cohort, the calibration curve (blue line) primarily resided below the ideal calibration line, suggesting the propensity of the model to overpredict positive outcomes. Nevertheless, as the mean predicted probability approached unity, the calibration of the model improved, converging toward the ideal calibration line. In the temporal validation cohort, the calibration curve (orange line) was mostly above the ideal calibration line, reflecting an underestimation of positive outcomes by the model. However, as the mean predicted probability increased, the calibration became more accurate, moving nearer to the ideal calibration line. The calibration curve for the external validation cohort (magenta line) significantly underpredicted in the intermediate probability range (0.4-0.7), subsequent calibration techniques applied to each cohort, as presented in Multimedia Appendix 7, demonstrated substantially improved calibration performance. Specifically, after applying calibration method using isotonic regression, the model showed enhanced generalizability across all datasets, with the calibrated curves exhibiting better alignment with the ideal calibration line. These results suggest that while the initial predictions may show some deviation from perfect calibration, appropriate postprocessing techniques can effectively address these discrepancies, supporting the model’s robust performance across different populations.

**Figure 4:**
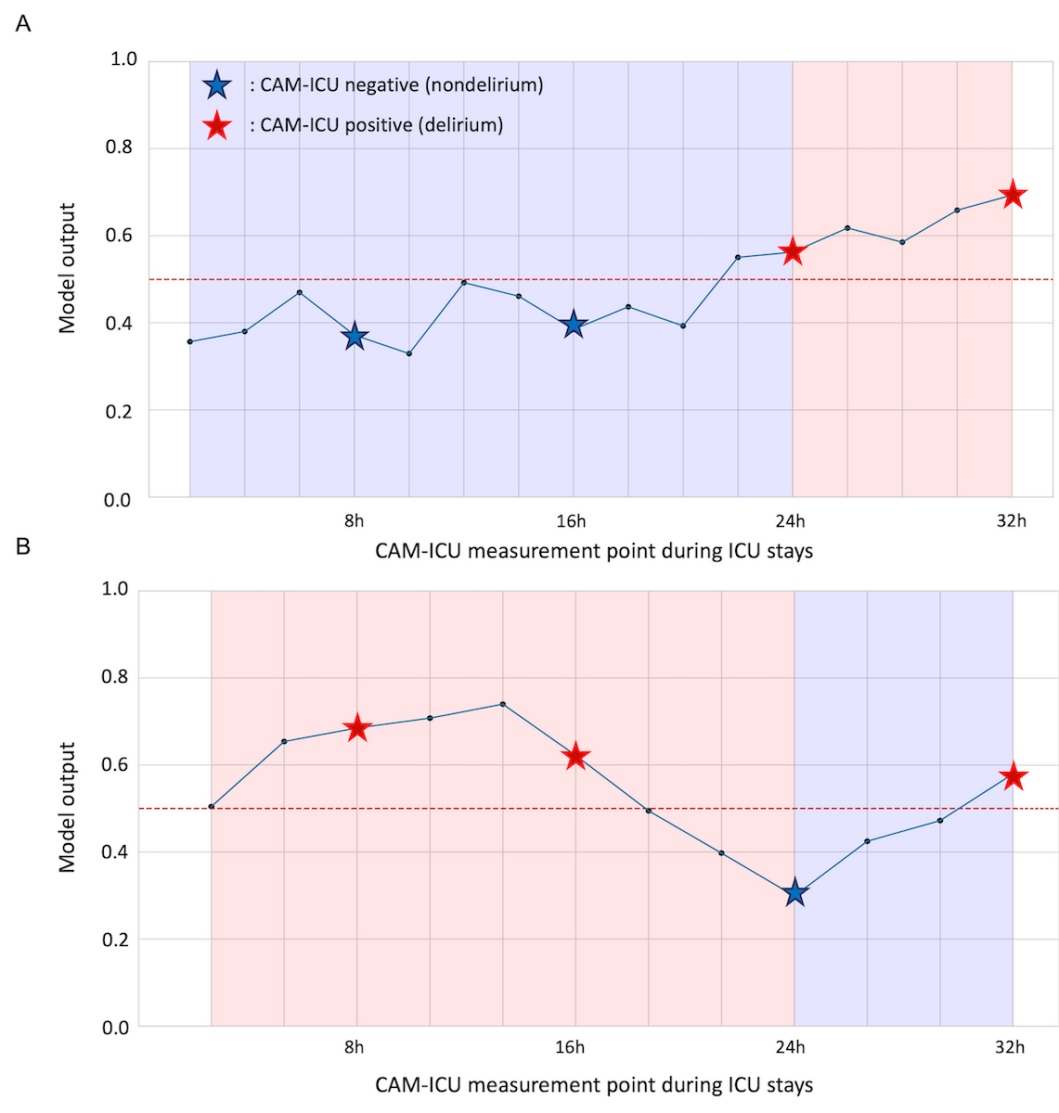
Temporal trends for model prediction during the intensive care unit stay. Confusion Assessment Method for the ICU (CAM-ICU) records of selected patients in the (A) internal validation cohort and (B) external validation cohort are shown. Red stars represent instances with positive evaluation results, indicating the presence of delirium. Blue stars represent instances with negative evaluation results, indicating the absence of delirium. Black dots denote the time at which no delirium evaluation was performed, precluding the acquisition of ground truth data. The red dashed line delineates the prediction threshold of the model and demarcates the boundary between predicted delirium and nondelirium statuses. ICU: intensive care unit.

In general, the prediction score concomitantly increased with positive CAM-ICU results, signaling the onset of delirium (Figure 4). Conversely, the prediction score remained low with negative CAM-ICU results, which was consistent with the absence of delirium. These findings suggested that our delirium prediction model could detect the onset of delirium in real time, even among patients who initially presented with no symptoms.

**Figure 5:**
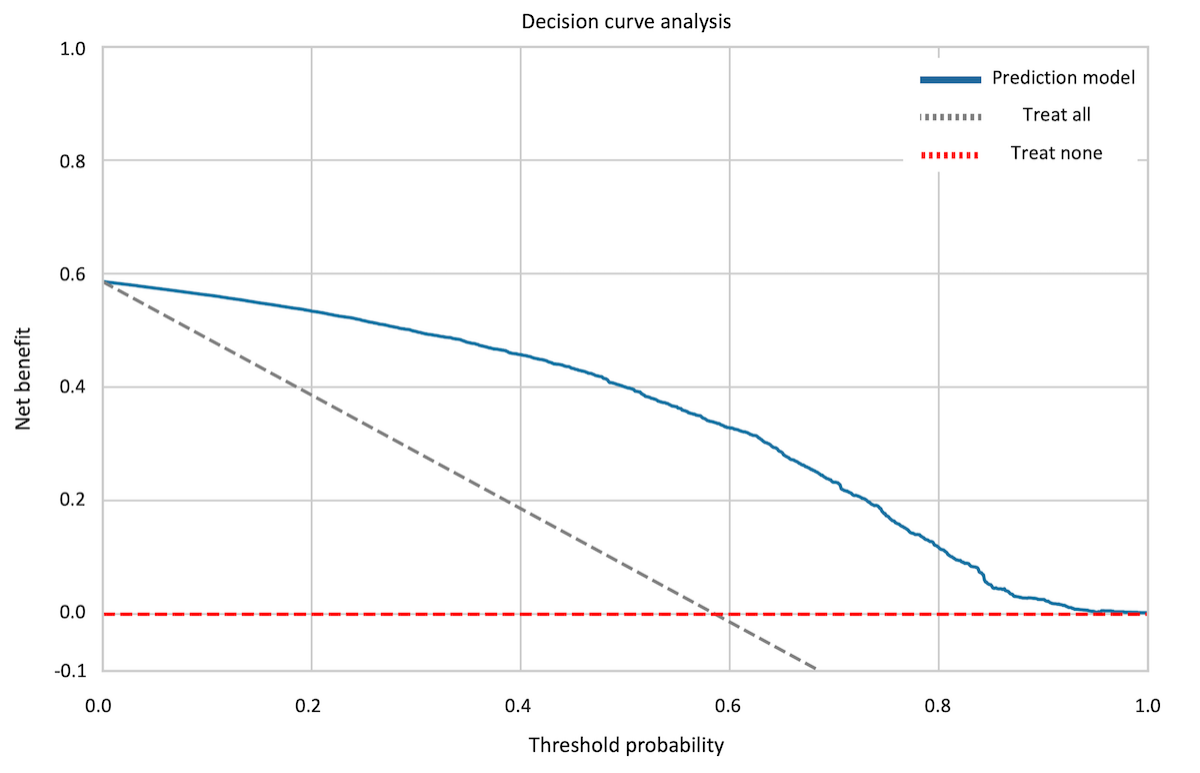
Quantified net benefit based on the threshold probability. Decision curve analysis shows the benefits and risks of decision-making regarding delirium using the delirium prediction model. The x-axis represents the threshold probability, whereas the y-axis represents the net benefit. The curve denotes the net benefit of using the delirium prediction model as compared to other clinical strategies (“treat all” and “treat none”). The data indicate that the delirium prediction model yields clinical benefit in clinical decision-making within all critical probability ranges.

Figure 5 shows the decision curve analysis for the prediction model in which the net benefit is plotted against various probability thresholds. The model presented a net benefit across a substantial spectrum of thresholds. Notably, the model began to yield a greater net benefit at a low threshold, compared with uniform treatment strategies (“treat all” and “treat none”), maintaining this advantage up to a threshold probability of approximately 0.6. This suggested the potential utility of the model in clinical decision-making, particularly when a lower probability was sufficient to warrant intervention. At no point within the examined threshold probabilities did the “treat all” strategy achieve a higher net benefit, underscoring the superiority of an individualized approach based on the model’s predictions. The analysis supported the application of the model in clinical settings, suggesting that it could enhance decision-making processes and potentially improve patient outcomes as compared to more generalized treatment strategies.

### Evaluation of Input Variables

**Figure 6:**
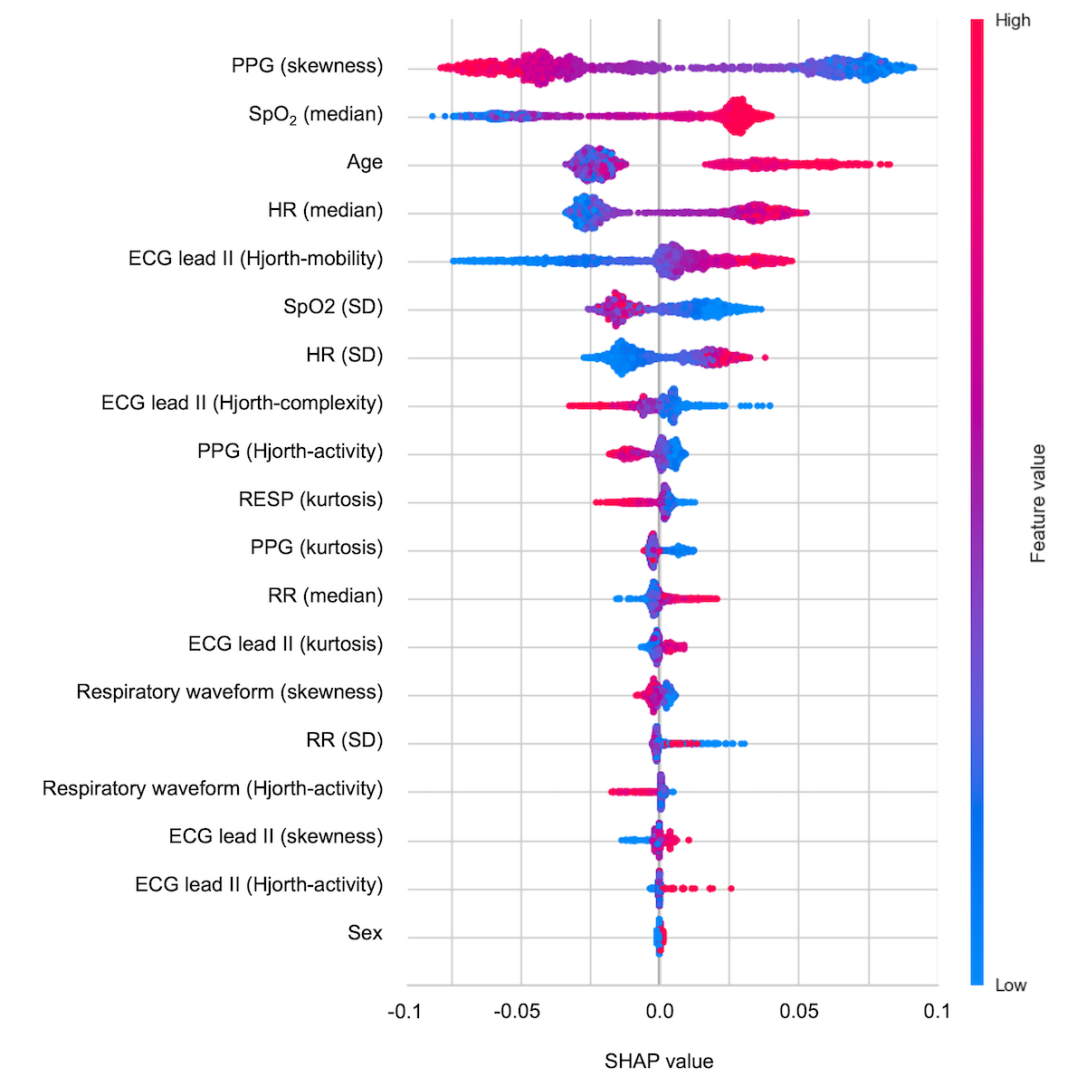
Force plot of Shapley additive explanations (SHAP) values in the validation set from the random forest model. The contributions of individual variables to the model’s predictions are identified and quantified using SHAP values. Age is the most significant predictive factor. The plot shows the substantial predictive value of various vital sign metrics and waveform variability indexes. ECG: electrocardiogram; HR: heart rate; PPG: photoplethysmogram; RR: respiratory rate; SHAP: Shapley additive explanations; SpO2: Oxygen saturation.

A comprehensive analysis with Shapley additive explanations values was performed to evaluate the predictive contributions of individual features in our model. On the basis of data from the internal validation cohort, age was one of the most influential predictors for the model's predictions, which aligns with existing clinical knowledge (Figure 6). Beyond age, our analysis revealed that a diverse array of indices derived from vital sign measurements and waveform variability significantly contributed to the predictive capacity of the model. These findings underscored the multifaceted and integrative nature of the predictive features harnessed by our algorithm.

## Discussion

### Principal Findings and Comparison to Prior Work

In this study, we developed a machine learning-based model for predicting delirium in real time during ICU stays using a compact set of routinely monitored variables. Our model employs ECG, PPG, and respiratory waveforms, addressing the challenge of data availability and aiming to develop a concise and broadly applicable model for predicting delirium.

Delirium remains a prevalent issue among ICU patients, often prolonging ICU stays and increasing mortality rates [55]. Despite being critical for delirium assessment, the CAM-ICU results are not regularly evaluated in several ICUs [56]. Given its high accuracy and ease of application, our model can substantially contribute to early delirium detection and prevent further patient deterioration. Even in ICUs where CAM-ICU results are regularly assessed, our model can identify at-risk patients during typically unassessed 8-hour intervals, supporting continuous monitoring.

Our model offers several advantages over existing delirium prediction tools. Although previous models such as PRE-DELIRIC, E-PRE-DELIRIC, and DYNAMIC-ICU show good initial performance [9,10,57], a recent study found that their performance dropped in other validation studies [58]. The detailed comparisons are provided in Table 4.

**Table 4:** Comparison of developed model with existing prediction tools.

	PRE-DELIRIC	DYNAMIC-ICU	E-PRE-DELIRIC	Our model
Algorithm	Logistic regression	Logistic regression	Logistic regression	Random forest
Dynamic features included	No	No	Yes (partially)	Yes
Variable measurement frequency	Once (admission)	Once (admission)	Once (admission), each laboratory test	Routine (continuous)
Model performance	Development cohort: 0.87 (95% CI 0.85-0.89), test cohort: 0.89 (95% CI 0.86-0.92)	Development cohort: 0.907 (95% CI 0.871-0.944), validation cohort: 0.900 (95% CI 0.858-0.941)	Development cohort: 0.76 (95% CI 0.73-0.78), validation cohort: 0.75 (95% CI 0.71-0.79)	Internal validation cohort: 0.83, external validation cohort: 0.84, temporal validation cohort: 0.73
Features	Age, APACHE^a^-II score, admission group, coma, infection, metabolic acidosis, use of sedatives, use of morphine, urea concentration, and urgent admission	History of chronic diseases, hearing deficits, infection, higher Apache II scores, use of sedatives and analgesics, indwelling catheter, and sleep disturbance	Age, history of cognitive impairment, history of alcohol abuse, blood urea nitrogen, admission category, urgent admission, mean arterial pressure, use of corticosteroids, and respiratory failure	Age, sex, ECG^b^-derived features (activity, complexity, mobility, kurtosis, skewness), PPG^c^-derived features (activity, kurtosis, skewness), respiratory waveform-derived features (activity, kurtosis, skewness), HR^d^ (median, SD), RR^e^ (median, SD), and SpO_2_^f^ (median, SD)

^a^APACHE: acute physiology and chronic health evaluation.

^b^ECG: electrocardiogram.

^c^PPG: photoplethysmogram.

^d^HR: heart rate.

^e^RR, respiratory rate.

^f^SpO_2_: oxygen saturation.

In contrast, our model demonstrated robust performance across both external and temporal validation methods. First, in external validation, the model maintained high performance (AUROC: 0.84 and AUPRC: 0.77) even when applied to a completely different patient population. Notably, the model was trained and developed using data from medical and surgical ICUs, whereas the external validation was conducted on trauma ICU patients, underscoring the model’s robustness across diverse clinical settings. Second, in temporal validation, which assesses stability over time, the model continued to exhibit strong performance (AUROC: 0.73 and AUPRC: 0.85). These results strongly indicate that our model maintains consistent predictive capability across varied patient populations and temporal changes, highlighting its potential for broad clinical applicability.

### Continuous Monitoring Approach and Clinical Utility

A key distinction of our approach is the use of continuous real-time data from ECG, PPG, and respiratory waveforms, unlike existing models that rely on static variables collected at specific time points. These signals are routinely monitored for most ICU patients, allowing for continuous data collection. Importantly, they can be measured noninvasively, minimizing the risk of adverse effects even if additional measurements are required. This characteristic is crucial, as it enables our model to be potentially implemented for a broad spectrum of ICU patients. The continuous nature of these measurements also allows our model to provide real-time, ongoing risk assessment, potentially capturing subtle physiological changes that might precede the onset of delirium. These features collectively enhance not only the model’s predictive capabilities but also its potential for widespread clinical implementation in diverse ICU settings. This allows for dynamic risk assessment throughout a patient’s ICU stay, potentially capturing subtle physiological changes preceding delirium onset that static models might miss. The efficacy of this dynamic risk assessment is well illustrated in Figure 4, which depicts the temporal progression of the model’s risk score. The figure incorporates star-shaped markers representing actual CAM-ICU assessments, with blue and red stars indicating negative and positive delirium assessments, respectively. Given that delirium assessments are not conducted hourly or in real time, the intervening data points represent the model’s computed risk scores. Notably, the model demonstrates an increase in risk scores as it approaches time points where positive delirium assessments were made, and conversely, a gradual decrease in risk scores preceding negative assessments. This inverse relationship between the model’s risk scores and the proximity to actual delirium occurrences or nonoccurrences underscores the clinical utility of the model in delirium prediction and risk assessment. By incorporating multiple physiological waveforms, our model extends beyond previous studies that found associations between individual parameters (such as HRV) and delirium but did not develop predictive models [59]. Real-time monitoring data significantly enhances the delirium prediction capacity. These waveforms offer several advantages in predicting delirium because they can reflect valuable information about the autonomic nervous system and its instability related to delirium. For instance, HRV, a well-established marker of autonomic nervous system function, can be derived from PPG and ECG data, with reduced HRV being associated with autonomic nervous system dysregulation [32-34,60]. HRV metrics provide a window into the balance between sympathetic and parasympathetic activities, and abnormalities of this balance are linked to delirium [61,62]. With respect to respiratory waveforms, their association with sedatives commonly used in ICUs is particularly important. Sedatives can alter respiratory function, and these alterations can be captured in respiratory waveforms. Monitoring of respiratory impedance becomes crucial, as altered respiratory patterns can be both a cause and a consequence of sedative use. Moreover, sedation is a well-known risk factor for delirium [9,63,64], further emphasizing the need for careful respiratory monitoring in sedated patients. As shown in our results, PPG-derived variables, SpO_2_, and age are among the most important predictors of delirium. The relationship between delirium and physiological measures, such as PPG and SpO_2,_ is complex and multifaceted. Previous studies did not directly link delirium to PPG or SpO_2_ measurements; however, analysis of the relationship between PaO_2_/FiO_2_ (a measure of pulmonary oxygenation) and delirium revealed a nonlinear relationship, suggesting that oxygenation status may influence delirium risk under certain conditions [65].

Age has been widely recognized as a major risk factor for the development of delirium [66]. The findings of our study corroborate this, demonstrating that age is a primary determinant in the incidence of delirium, which is consistent with prior knowledge. Therefore, we evaluated differences in the model performance according to various age groups (Multimedia Appendix 8). We considered the entire population, individuals aged 40 to 60 years, and those aged ≥60 years to assess the model’s performance across different age groups. The model showed consistent performance across these groups, with an AUROC of 0.82 for the entire population, 0.81 for individuals aged 40 to 60 years, and 0.83 for those aged ≥60 years, and with an AUPRC of 0.62 for the entire population, 0.63 for individuals aged 40 to 60 years, and 0.68 for those aged ≥60 years. These findings underscore the model’s robustness, irrespective of the age group, thereby highlighting the model’s reliability across diverse clinical scenarios. Although delirium generally occurs more frequently in older patients, it can also affect younger populations, with an incidence of 4.4% and up to 14% in high-risk groups [21]. Age continues to serve as a significant predictor of delirium onset, and our findings highlight the potential of prediction models constructed using ECG, PPG, and respiratory waveforms for any age group. Hence, variables derived from such waveforms provide valuable information beyond age.

Although traditional correlation analysis provides a useful baseline, it may not fully capture the intricate relationships in our data. Our RF model’s ability to identify these features as important, despite their low linear correlations, suggests that it is leveraging more complex, possibly nonlinear relationships to improve prediction accuracy.

Our tree-based model offers interpretability, addressing the “black box” limitation often associated with artificial intelligence in health care. This is crucial in medical settings where understanding the reasoning behind predictions is as important as the predictions themselves. The RF algorithm we used constructs multiple decision trees, each contributing to the final prediction. This approach enhances predictive accuracy while providing insights into feature importance and decision boundaries.

Examination of our model’s decision trees (Multimedia Appendix 9) reveals the hierarchical importance of various features in predicting delirium, such as age, vital signs, and waveform-derived features. This transparency allows medical professionals to align the model’s reasoning with their clinical judgment and established medical knowledge. It also facilitates identification of potential biases or unexpected patterns, enabling continuous refinement and validation of the model. This is particularly important in critical care, where patient conditions and treatment protocols can change rapidly.

Our decision curve analysis demonstrated the clinical utility of our delirium prediction model. The model consistently showed a positive net benefit exceeding both “treat all” and “treat none” strategies across all threshold probabilities. This indicates that our model provides value in clinical decision-making regardless of risk tolerance levels, offering a more nuanced approach to delirium prediction. It can help clinicians avoid both overtreatment and undertreatment by effectively identifying low-risk patients who may not need intensive preventive measures and high-risk patients who might otherwise be overlooked. The model’s potential to improve patient outcomes through more accurate risk stratification is highlighted by its positive net benefit across all thresholds.

The ultimate goal of the delirium prediction model is seamless integration into clinical workflows via electronic health record systems and bedside monitoring devices. This research serves as a foundational step in a broader implementation strategy. Prospective validation studies are necessary before clinical deployment, focusing on real-time Shapley additive explanations for clinicians’ trust and understanding. Clear ethical guidelines must be established to prevent overdiagnosis and maintain appropriate human oversight. Robust model generalizability must be achieved via rigorous validation procedures. An automated alert system with actionable clinical recommendations must be tested. Building upon this foundational research, our future studies will focus on validating the model’s real-world effectiveness and practical utility in clinical settings. The next phase of research will involve obtaining necessary regulatory approvals and ensuring compliance with safety and performance standards required for critical care decision support tools. This research reflects a commitment to developing robust, clinically validated tools for early delirium detection and improving patient outcomes in ICUs.

### Limitations

Our study has some limitations that should be addressed in future research. First, the external validation of our model was limited to use of data from a single institution; therefore, diverse patient populations across various health care settings may not be fully represented. However, the patient populations used in the model development environment and the external validation environment are from completely different environments, ICUs, and completely independent hospitals. We confirmed that the model performance was maintained in these environments.

Second, the retrospective nature of the study, conducted at a local ICU, may have introduced selection bias, which we attempted to mitigate by excluding incomplete data and validating the remaining data. This approach may limit the model’s applicability in scenarios with missing data points. The features used as input to our model are less susceptible to these issues because they are routinely collected in the ICU; however, future studies could consider adopting multiple imputation methods to enhance model performance and stability with incomplete data, as demonstrated by Rahmatinejad et al [67].

Third, while our temporal validation approach used systematic quarterly intervals (Q1-Q4) to account for seasonal variations, we acknowledge that additional standardization could enhance future studies. For example, future research could benefit from prespecifying the exact proportion of data to be used for validation or establishing multi-year validation periods. Nevertheless, our current approach using quarterly divisions over approximately a year of data after model development provided sufficient temporal range to assess the model’s performance across seasonal variations and evolving clinical practices.

Finally, although our model focuses on predicting the onset of delirium, it does not address other important aspects, such as the duration or severity of delirium episodes. Expanding the model to predict these additional factors would significantly enhance its clinical utility. For instance, differentiating between patients likely to develop mild, short-term delirium versus those at risk of severe, prolonged episodes could greatly inform treatment decisions and resource allocation in ICU settings. However, the evaluation of delirium in ICUs is conducted by nurses, and data on the duration of the condition cannot be found in hospital records. Due to the nature of supervised learning, when there is a clear correct answer, the learning proceeds through it, so there is a clear limit to the practical implementation.

By exploring different data handling techniques, expanding validation to diverse clinical environments, assessing real-world clinical impact, and extending the model’s predictive capabilities, we can work toward more robust and widely applicable delirium prediction tools. These efforts have the potential to significantly improve patient care and outcomes in ICUs.

### Conclusions

In conclusion, we developed a machine learning model for real-time delirium prediction in ICUs by using a concise set of input variables, including physiological waveforms such as ECG, PPG, and respiratory patterns. Our model not only identified age as a significant predictor but also highlighted the substantial predictive value of these waveforms, independent of age. These waveforms provide critical insights into patients’ conditions and offer potential for early delirium detection. Overall, our model exhibits high performance in both internal and external validation and has broad applicability across health care settings, potentially contributing to the development of effective early intervention strategies to improve patient outcomes.

## Appendix

Multimedia Appendix 1 Technical and methodological details.

Multimedia Appendix 2 Comparison of normal and abnormal waveform morphology.

Multimedia Appendix 3 Schematic of data processing for model input features.

Multimedia Appendix 4 Performance comparison of tree-based machine learning models.

Multimedia Appendix 5 Quarterly and overall model performance metrics.

Multimedia Appendix 6 Correlation heatmap of predictor variables with delirium outcome.

Multimedia Appendix 7 Calibration plots across training and validation cohorts.

Multimedia Appendix 8 Model performance across different age groups.

Multimedia Appendix 9 Sample decision tree from random forest model.

## Abbreviations

AUPRC: area under the precision-recall curve
AUROC: area under the receiver operating characteristic curve
CAM-ICU: Confusion Assessment Method for the ICU
ECG: electrocardiogram
HRV: heart rate variability
ICU: intensive care unit
IRB: institutional review board
LightGBM: light gradient boosting model
PPG: photoplethysmogram
RF: random forest
TRIPOD+AI: Transparent Reporting of a multivariable prediction model for Individual Prognosis or Diagnosis+Artificial Intelligence

## Data Availability

The datasets generated or analyzed during this study are not publicly available due to hospital policy. Data may be used only after institutional review board review of the research plan and data review board approval for data use. The codes used for model development are available in our GitHub repository [68].
