# Supplementary material for: Development and Validation of a Machine Learning Model for Early Prediction of Delirium in Intensive Care Units Using Continuous Physiological Data: Retrospective Study
Source: J Med Internet Res. 2025 Apr 2;27:e59520. doi: 10.2196/59520 (PMC12004028; doi:10.2196/59520)
Supplement: Multimedia Appendix 1 [file jmir_v27i1e59520_app1.docx]

## Supplementary Information

**Development and Validation of a Machine Learning Model for Early Prediction of Delirium in Intensive Care Units Using Continuous Physiological Data : a retrospective study**

Chan Min Park, MS^1^; Changho Han, MD, MS^1^; Su Kyeong Jang, MS^2^; Hyungjun Kim, MS^2^; Sora Kim, RN, BSN^3^; Byung Hee Kang, MD^4^; Kyoungwon Jung, MD, PhD^3,4^; Dukyong Yoon, MD, PhD^1,5,6†^

^1^ Department of Biomedical Systems Informatics, Yonsei University College of Medicine, Seoul, Republic of Korea

^2^ BUD.on Inc., Seoul, Republic of Korea

^3^ Ajou University Hospital Gyeonggi South Regional Trauma Center, Suwon, Republic of Korea

^4^ Division of Trauma Surgery, Department of Surgery, Ajou University School of Medicine, Suwon, Republic of Korea

^5^ Institute for Innovation in Digital Healthcare (IIDH), Severance Hospital, Seoul, Republic of Korea

^6^ Center for Digital Health, Yongin Severance Hospital, Yonsei University Health System, Yongin, Republic of Korea

**Corresponding Author:**

Dukyong Yoon, MD, PhD

Department of Biomedical Systems Informatics, Yonsei University College of Medicine

Seoul, Republic of Korea

Phone: +82 31-5189-8450; Fax: +82 31-5189-8566

E-mail: dukyong.yoon@yonsei.ac.kr

**Quality control from two healthcare institutions**

To ensure consistency and reliability of data across the two healthcare institutions, we implemented a rigorous standardization and quality control process. We confirmed that both hospitals employed identical biosignal collection systems [1]. This method involves transmitting raw data from the ICU bedside monitor to a server within the hospital, encoding it, and converting it into a database. We verified that the frequencies of the raw data were consistent across both institutions: 500 Hz for ECG, 125 Hz for PPG, and 62 Hz for respiratory waveforms.

Prior to feature extraction, we implemented a comprehensive preprocessing pipeline to enhance signal quality and remove artifacts:

Baseline Wander Removal: We applied a high-pass filter with a cutoff frequency of 0.5 Hz to remove low-frequency baseline drift from the ECG and PPG signals. This step is crucial for maintaining signal stability and improving the accuracy of subsequent analyses.

Motion Artifact Removal: We employed an adaptive filter technique to detect and remove motion artifacts from the ECG, PPG, and respiratory waveforms. This process involved identifying sudden, large-amplitude changes in the signal that are characteristic of motion artifacts and replacing these segments with interpolated values based on surrounding data points.

Bandpass Filtering: We applied a Butterworth bandpass filter to each signal type:

1. ECG: 0.5-40 Hz to preserve the primary ECG components while removing high-frequency noise.
2. PPG: 0.5-10 Hz to retain the main PPG signal.
3. Respiratory waveforms: 0.05-1 Hz to focus on the frequency range of normal breathing patterns.

These filters were designed to remove noise while preserving the physiologically relevant components of each signal.

Standardization: Following the initial preprocessing steps, we applied standardization to normalize the amplitude of the signals. This was done by subtracting the mean and dividing by the standard deviation of each signal segment. This step is crucial for ensuring that the subsequent feature extraction processes are not biased by differences in signal amplitude between patients or recording sessions.

After these preprocessing steps, we proceeded with feature extraction. We calculated Hjorth parameters (activity, mobility, and complexity) and shape features (kurtosis and skewness) for each signal type. These features provide information about the signal's time domain properties and morphology, respectively.

Hjorth Parameters:

The Hjorth parameters are time-domain measures that characterize signal properties. For a time series x(t), we calculated:

1. Activity: This represents the signal power, computed as the variance of the signal.

$$Activity=var(x\left( t \right))$$

2. Mobility: This represents the mean frequency of the signal.

$$Mobility=\sqrt{\frac{var\left( \frac{dx\left( t \right)}{dt} \right)}{var\left( x\left( t \right) \right)}}$$

Where dx/dt is the first derivative of the signal.

1. Complexity: This represents the change in frequency.

$$Complexity=\frac{Mobility\left( \frac{dx(t)}{dt} \right)}{Mobility(x\left( t \right))}$$

Shape Features:

a) Kurtosis: This measures the "tailedness" of the probability distribution of the signal.

$$Kurtosis=\frac{E[{(x-\mu)}^{4}]}{{(E[{(x-\mu)}^{2}])}^{2}}$$

Where E is the expected value, x is the signal, and μ is the mean of x.

b) Skewness: This measures the asymmetry of the probability distribution of the signal.

$$Skewness=\frac{E[{(x-\mu)}^{3}]}{{(E[{(x-\mu)}^{2}])}^{\frac{3}{2}}}$$

To mitigate the impact of extreme values and account for the inherently noisy nature of physiological signals, we calculated representative values (medians and standard deviations) over a 4-hour predictive window prior to delirium assessment for each feature. This approach ensures robust model operation by minimizing the influence of outliers.

To maximize data integrity, we excluded any data with missing values. This rigorous preprocessing and standardization pipeline was applied consistently to data from both healthcare institutions, ensuring a harmonized dataset for model development.

**Addressing Class Imbalance & Hyperparameter Tuning**

To address the class imbalance inherent in delirium prediction, we implemented several strategies in our Random Forest model. First, we employed a balanced class weighting approach, which automatically adjusts the importance of each class inversely proportional to its frequency in the input data. This method assigns higher importance to the minority class (delirium cases), effectively compelling the model to pay more attention to the underrepresented class [2].

For node splitting in the decision trees, we utilized the entropy criterion rather than the default Gini impurity measure. This choice was based on evidence suggesting that entropy can perform better on imbalanced datasets [3]. Additionally, we enabled bootstrap sampling, which creates different subsets of the data for each tree in the forest. This technique can help mitigate the impact of class imbalance by providing varied representations of the dataset to different trees.

Following these initial settings, we conducted an automated hyperparameter tuning process using a random grid search strategy with 10-fold cross-validation. This process explored various combinations of hyperparameters, with the objective of maximizing precision as our chosen performance metric.

The hyperparameter optimization process resulted in the following settings: a minimum impurity decreases of 0.0005, a minimum of 5 samples required to be at a leaf node, a minimum of 9 samples required to split an internal node, a maximum tree depth of 3, and a total of 50 trees in the forest. These carefully tuned parameters help prevent overfitting, which is particularly crucial in imbalanced datasets to avoid creating branches that only capture a small number of minority class samples.

**Net benefit equation for Decision Curve Analysis**

$$Net benefit=True positives-w*\frac{False positives}{Total population}$$

where $w$ is typically defined as

$$w=\frac{Threshold}{1- Threshold}$$

However, this case assumes that the costs of false positives and false negatives are the same. In the case of delirium, the traditional *w* can be redefined by referring to a previous report, which showed that the cost of false positives is 0.519 times that of false negatives [4]. $w'$ is defined as follows:

$$w^{'}=0.519 * \frac{Threshold}{1- Threshold}$$

Now, new net benefit is defined as follows:

$$New Net benefit=True positives-w^{'}*\frac{False positives}{Total population}$$

$$=True positives-(0.519*\frac{Threshold}{1- Threshold}*\frac{False positives}{Total population})$$

**Checklist for TRIPOD+AI Guideline**


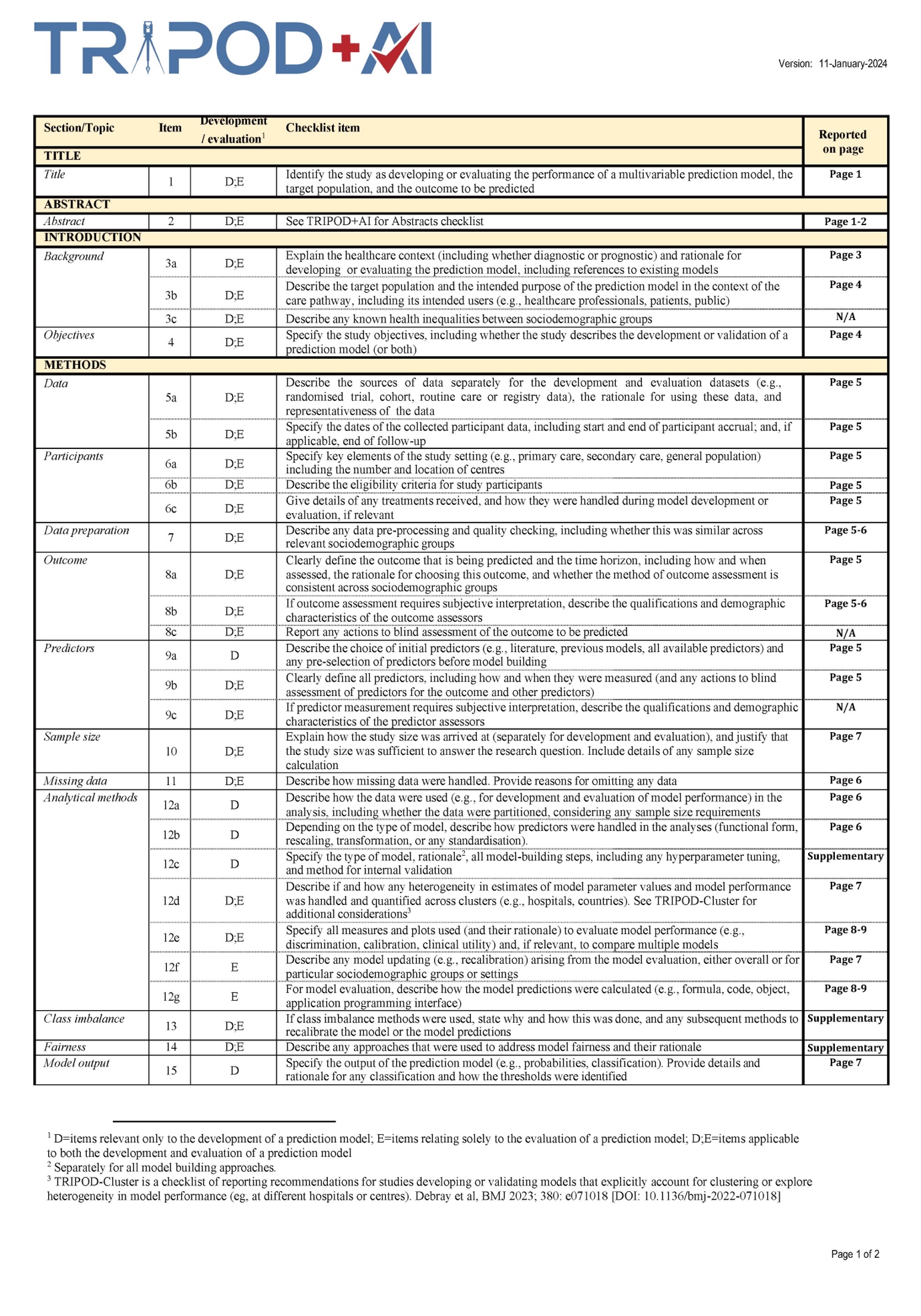

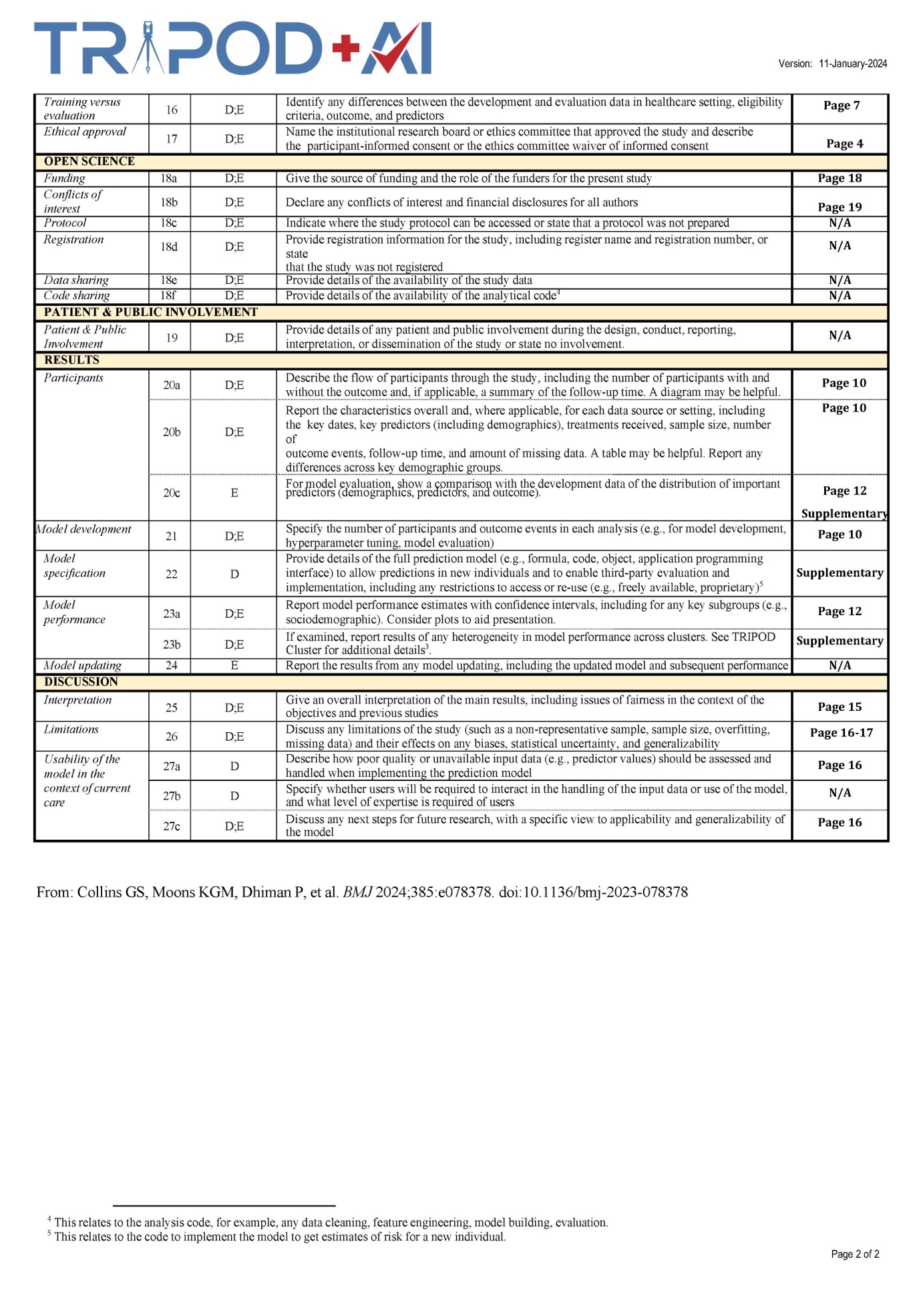


**Checklist for Guidelines for Developing and Reporting Machine Learning Predictive Models in Biomedical Research**

| Item number | Section | Topic | Checklist Item | Page |
| --- | --- | --- | --- | --- |
| 1 | Title | Nature of study | Identify the report as introducing a predictive model | Page 1 |
| 2 | Abstract | Structured summary | Background  Objectives  Data sources  Performance metrics of the predictive model or models, in both point estimates and confidence intervals  Conclusion including the practical value of the developed predictive model or models | Page 1,2 |
| 3 | Introduction | Rationale | Identify the clinical goal Review the current practice and prediction accuracy of any existing models | Page 4 |
| 4 | Introduction | Objectives | State the nature of study being predictive modeling, defining the target of prediction Identify how the prediction problem may benefit the clinical goal | Page 4 |
| 5 | Methods | Describe the setting | Identify the clinical setting for the target predictive model.  Identify the modeling context in terms of facility type, size, volume, and duration of available data. | Page 5 |
| 6 | Methods | Define the prediction  problem | Define a measurement for the prediction goal (per patient or per hospitalization or per type of outcome). Determine that the study is retrospective or prospective. Identify the problem to be prognostic or diagnostic. Determine the form of the prediction model: (1) classification if the target variable is categorical, (2) regression if the target variable is continuous, (3) survival prediction if the target variable is the time to an event. Translate survival prediction into a regression problem, with the target measured over a temporal window following the time of prediction. Explain practical costs of prediction errors (eg, implications of underdiagnosis or overdiagnosis). Defining quality metrics for prediction models. Define the success criteria for prediction (eg, based on metrics in internal validation or external validation in the context of the clinical problem). | Page 5,6 |
| 7 | Methods | Prepare data for model building | Identify relevant data sources and quote the ethics approval number for data access.  State the inclusion and exclusion criteria for data.  Describe the time span of data and the sample or cohort size.  Define the observational units on which the response variable and predictor variables are defined.  Define the predictor variables. Extra caution is needed to prevent information leakage from the response variable to predictor variables.  Describe the data preprocessing performed, including data cleaning and transformation. Remove outliers with impossible or extreme responses; state any criteria used for outlier removal.  State how missing values were handled.  Describe the basic statistics of the dataset, particularly of the response variable. These include the ratio of positive to negative classes for a classification problem and the distribution of the response variable for regression problem.  Define the model validation strategies. Internal validation is the minimum requirement; external validation should also be performed whenever possible.  Specify the internal validation strategy. Common methods include random split, time-based split, and patient-based split.  Define the validation metrics. For regression problems, the normalized root-mean-square error should be used. For classification problems, the metrics should include sensitivity, specificity, positive predictive value, negative predictive value, area under the ROC curve, and calibration plot | Page 6 |
| 8 | Methods | Build the predictive model | For retrospective studies, split the data into a derivation set and a validation set. For prospective studies, define the starting time for validation data collection.  Identify independent variables that predominantly take a single value (eg, being zero 99% of the time). Identify and remove redundant independent variables. Identify the independent variables that may suffer from the perfect separation problem.^f^ Report the number of independent variables, the number of positive examples, and the number of negative examples. Assess whether sufficient data are available for a good fit of the model. In particular, for classification, there should be a sufficient number of observations in both positive and negative classes. Determine a set of candidate modeling techniques (eg, logistic regression, random forest, or deep learning). If only one type of model was used, justify the decision for using that model. Define the performance metrics to select the best model. Specify the model selection strategy. Common methods include K-fold validation or bootstrap to estimate the lost function on a grid of candidate parameter values. For K-fold validation, proper stratification by the response variable is needed. For model selection, include discussion on (1) balance between model accuracy and model simplicity or interpretability, and (2) the familiarity with the modeling techniques of the end user. | Page 6-8 |
| 9 | Results | Report the final model and performance | Report the predictive performance of the final model in terms of the validation metrics specified in the methods section. If possible, report the parameter estimates in the model and their confidence intervals. When the direct calculation of confidence intervals is not possible, report nonparametric estimates from bootstrap samples. Comparison with other models in the literature should be based on confidence intervals. Interpretation of the final model. If possible, report what variables were shown to be predictive of the response variable. State which subpopulation has the best prediction and which subpopulation is most difficult to predict. | Page 12 |
| 10 | Discussion | Clinical implications | Report the clinical implications derived from the obtained predictive performance. For example, report the dollar amount that could be saved with better prediction. How many patients could benefit from a care model leveraging the model prediction? And to what extent? | Page 13, 14 |
| 11 | Discussion | Limitations of the model | Discuss the following potential limitations:  • Assumed input and output data format  • Potential pitfalls in interpreting the model  • Potential bias of the data used in modeling  • Generalizability of the data | Page 18,19 |
| 12 | Discussion | Unexpected results during the experiments | Report unexpected signs of coefficients, indicating collinearity or complex interaction between predictor variables | Multimedia Appendix |

## References

1. Yoon D, Lee S, Kim TY, Ko J, Chung WY, Park RW. System for Collecting Biosignal Data from Multiple Patient Monitoring Systems. Healthc Inform Res. 2017 Oct;23(4):333-7. PMID: 29181244. doi: 10.4258/hir.2017.23.4.333.

2. Chen C, Liaw A, Breiman L. Using random forest to learn imbalanced data. University of California, Berkeley. 2004;110(1-12):24.

3. Raileanu LE, Stoffel K. Theoretical comparison between the gini index and information gain criteria. Annals of Mathematics and Artificial Intelligence. 2004;41:77-93.

4. MacLullich AM, Shenkin SD, Goodacre S, Godfrey M, Hanley J, Stiobhairt A, et al. The 4 'A's test for detecting delirium in acute medical patients: a diagnostic accuracy study. Health Technol Assess. 2019 Aug;23(40):1-194. PMID: 31397263. doi: 10.3310/hta23400.
