# Supplementary figures and images for: Development and Validation of a Machine Learning Model for Early Prediction of Delirium in Intensive Care Units Using Continuous Physiological Data: Retrospective Study
Source: J Med Internet Res. 2025 Apr 2;27:e59520. doi: 10.2196/59520 (PMC12004028; doi:10.2196/59520)

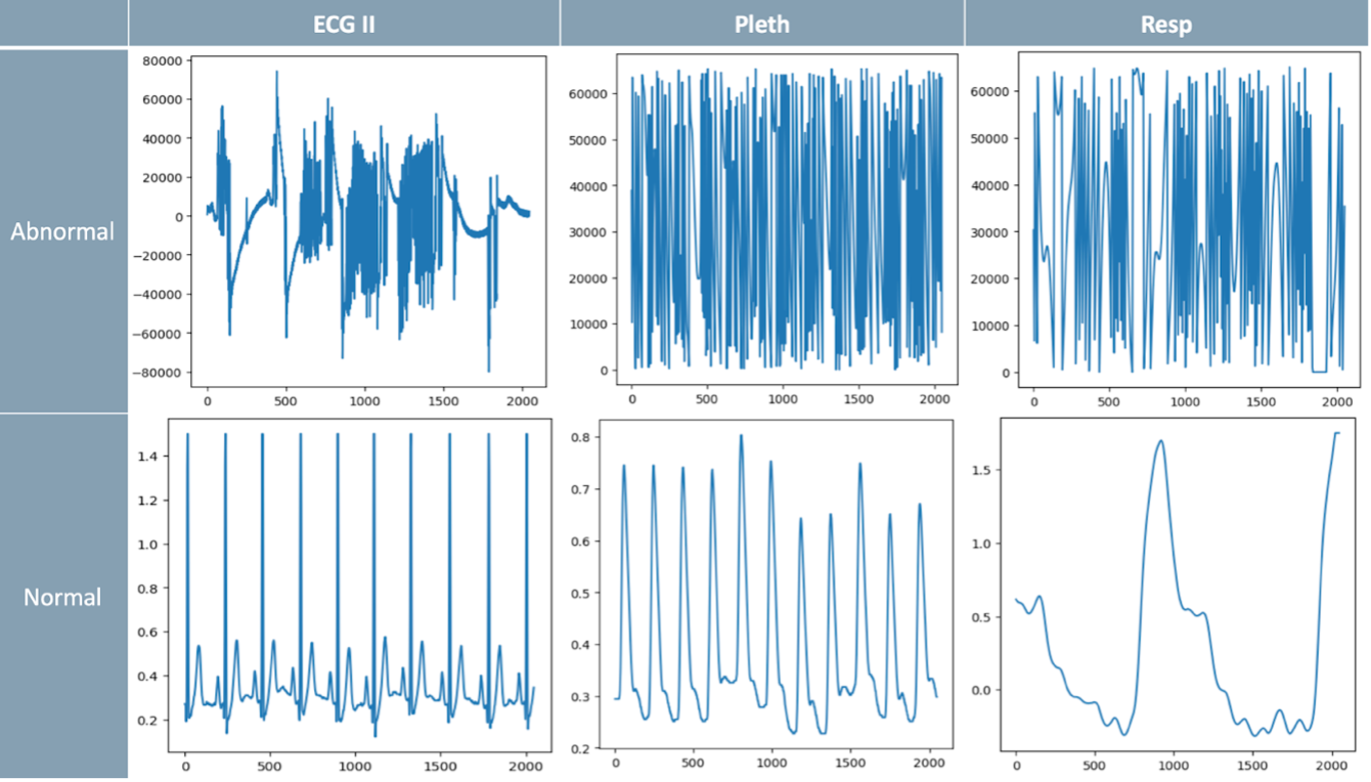

Supplement: Multimedia Appendix 2 [file jmir_v27i1e59520_app2.png]

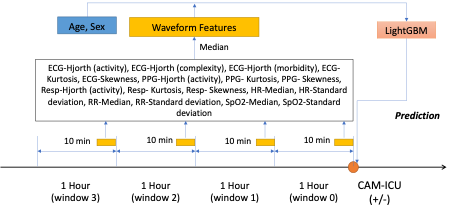

Supplement: Multimedia Appendix 3 [file jmir_v27i1e59520_app3.png]

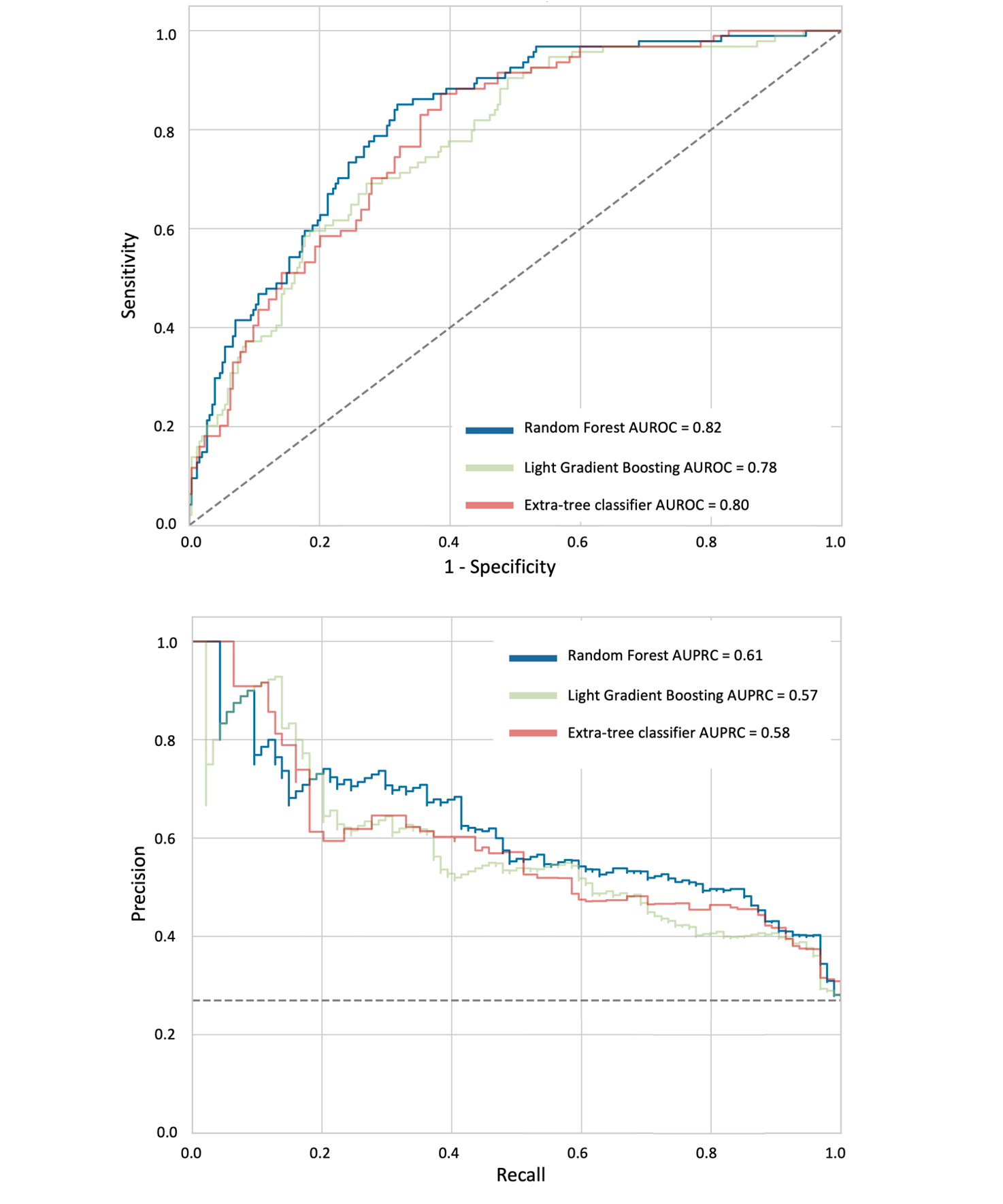

Supplement: Multimedia Appendix 4 [file jmir_v27i1e59520_app4.png]

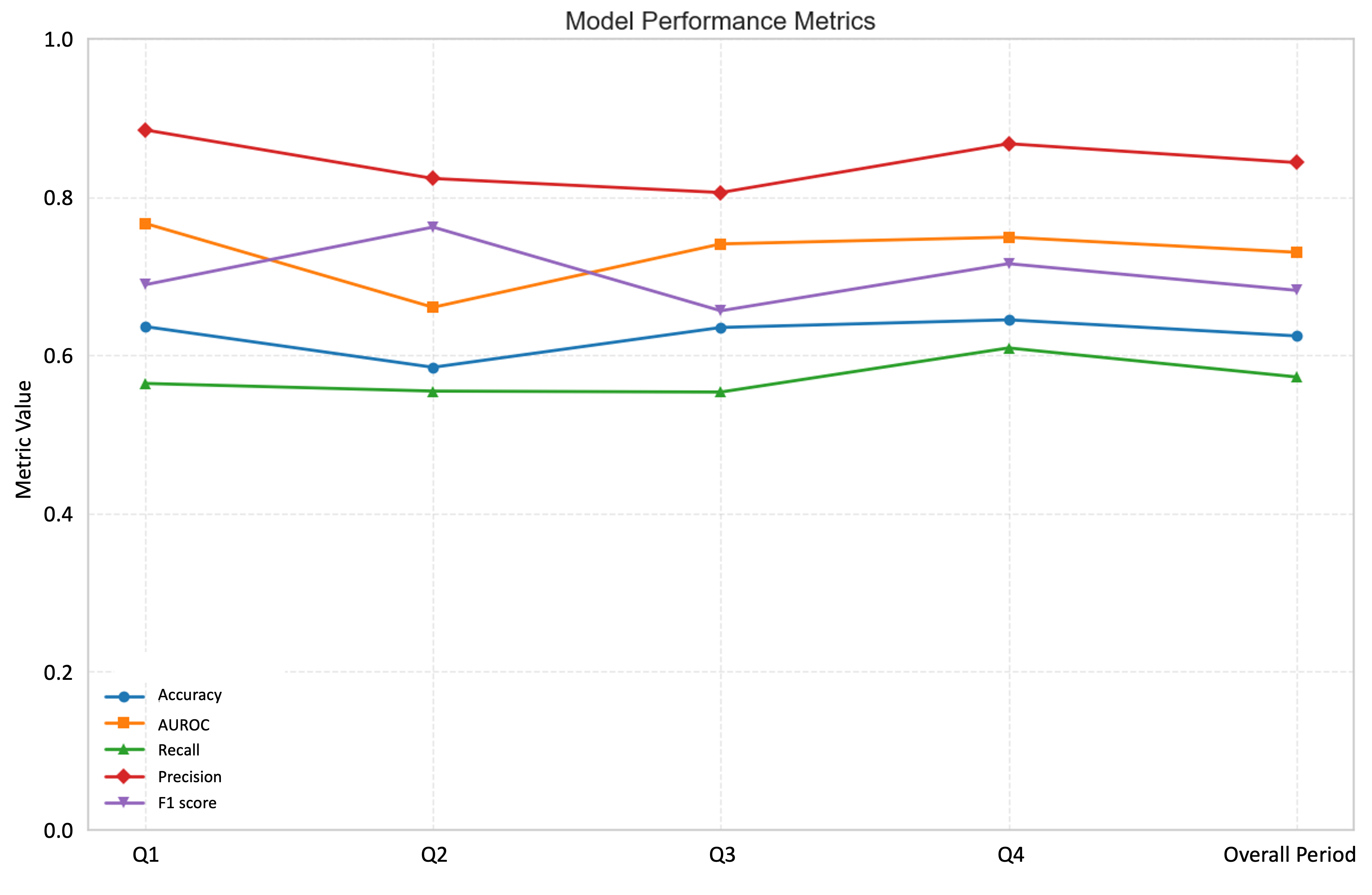

Supplement: Multimedia Appendix 5 [file jmir_v27i1e59520_app5.png]

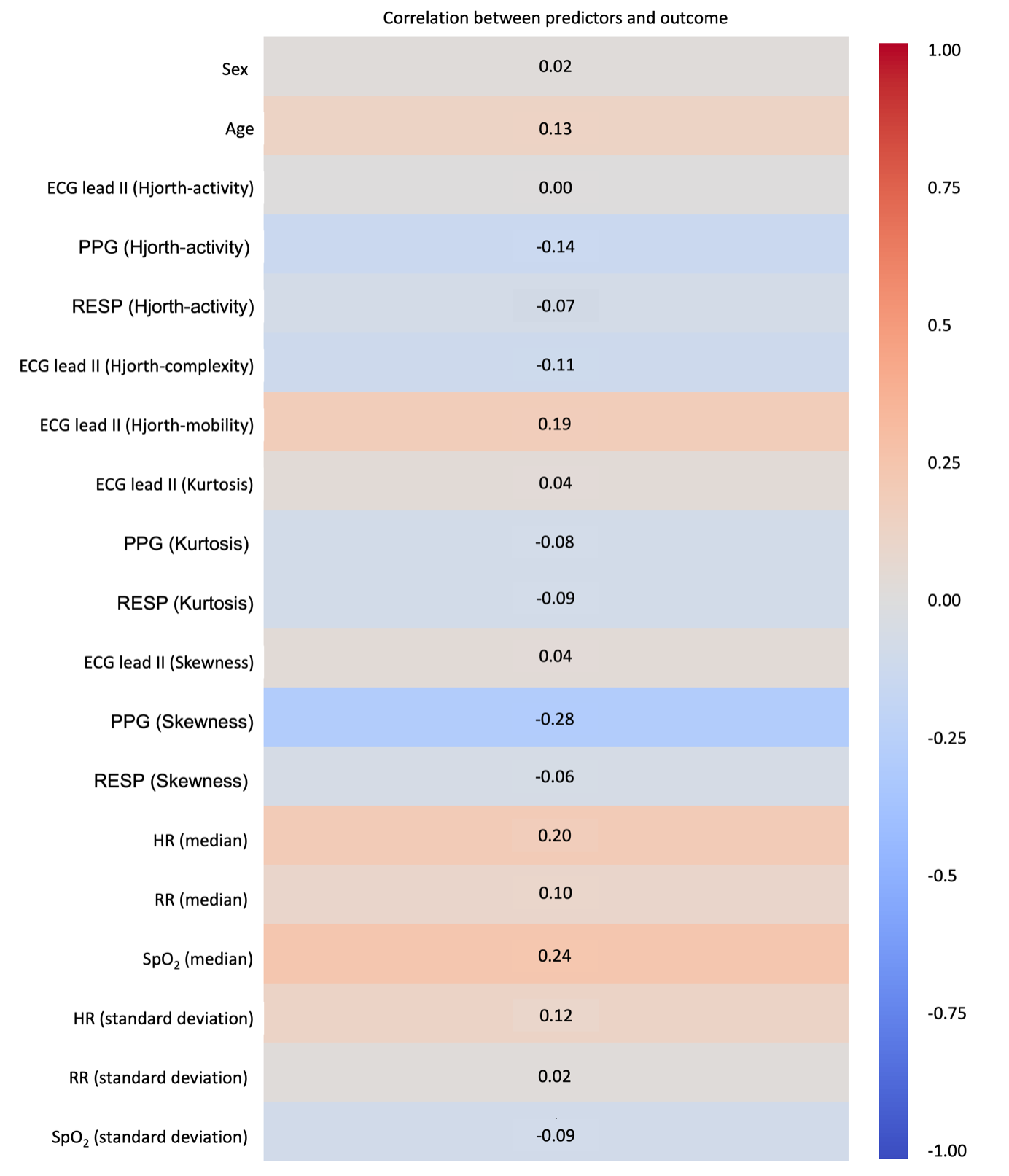

Supplement: Multimedia Appendix 6 [file jmir_v27i1e59520_app6.png]

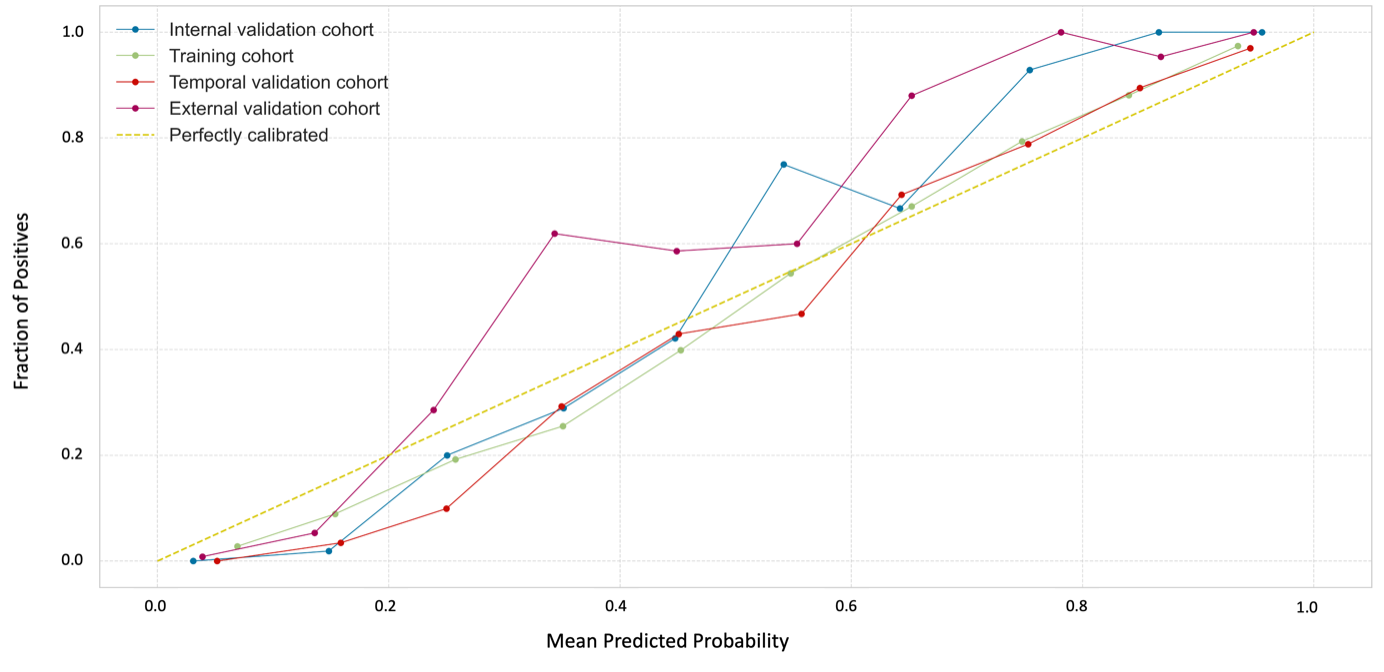

Supplement: Multimedia Appendix 7 [file jmir_v27i1e59520_app7.png]

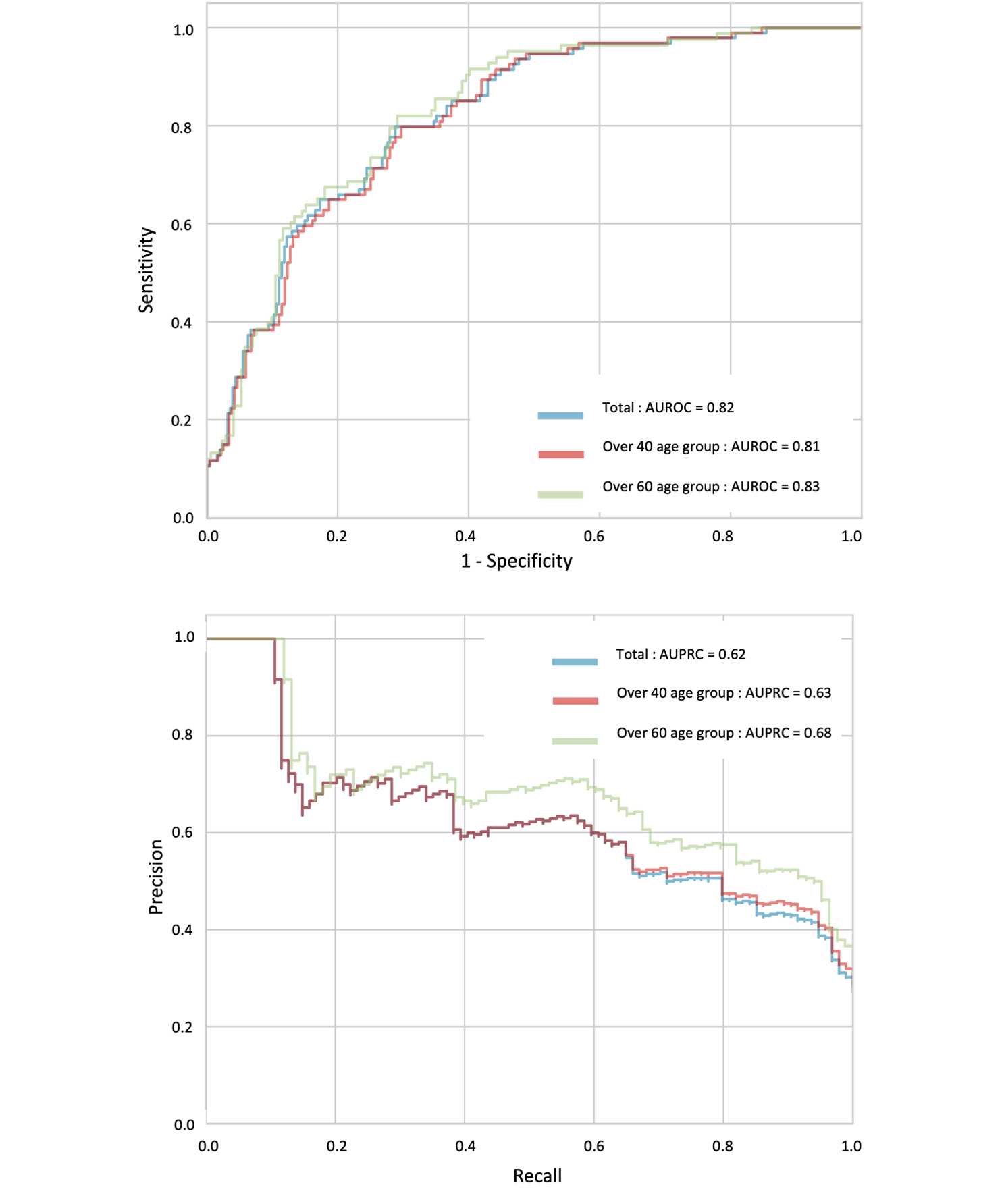

Supplement: Multimedia Appendix 8 [file jmir_v27i1e59520_app8.png]

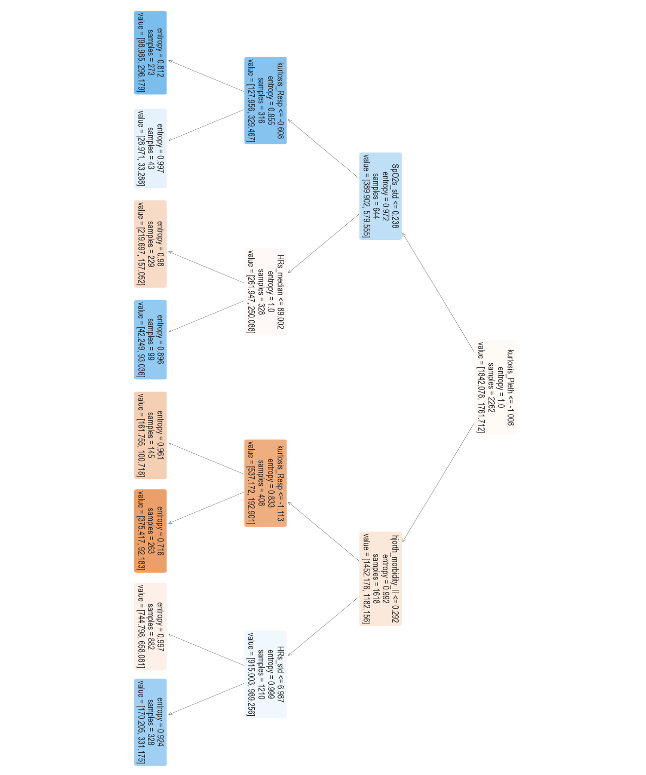

Supplement: Multimedia Appendix 9 [file jmir_v27i1e59520_app9.png]
